# The FORGENIUS Genomic Resources: New Genotyping Tools and Genomic Data for 23 Forest Tree Species and Their Genetic Conservation Units

**DOI:** 10.1111/1755-0998.70115

**Published:** 2026-03-03

**Authors:** Sara Pinosio, Francesca Bagnoli, Camilla Avanzi, Maria B. Castellani, Arcangela Frascella, Susan L. McEvoy, Sanna Olsson, Ilaria Spanu, Elia Vajana, the FORGENIUS Consortium, Marc Busuldu, Vega Garcia‐Segura, Ana Hernández‐Serrano, Sonia Hernando, Maurizio Mencuccini, Joan Prunera‐Olivé, Carla Rodrigo‐González, Adolfo Sanmartín‐Arévalo, Martí Solé‐Xanxo, Laura Wynne Stewart, Ana Cabanillas, Jonathan Feichter, Berthold Heinze, Akkin Semerci, Katrin Heer, Jill Sekely, Stuart A’Hara, Joan Cottrell, Eduardo Notivol, Christophe Gauvrit, Marie‐Gabrielle Harribey, Joan Hochet, Baptiste Laffitte, Matteo Nasuti, Annie Raffin, Thomas Francisco, Adélaïde Theraroz, Pierre‐Jean Dumas, Florence Jean, Nicolas Mariotte, Maurizio Marchi, Ricardo Alía, Francisco J. Auñón, Diana Barba, Maria Regina Chambel, Fernando del Caño, María Carmen García Barriga, José Manuel García del Barrio, Delphine Grivet, Rodrigo Pulido Sanz, Ana Iordan, Flaviu Popescu, Dragos Postolache, Daniel Suciu, Leena Yrjänä, Egbert Beuker, Henri Hämäläinen, Hanna‐Riikka Haurinen, Christian Mestre‐Runge, Lars Opgenoorth, Christian Reudenbach, Marko Bajc, Gregor Božič, Rok Damjanić, Natalija Dovč, Luka Krajnc, Marija Kravanja, Gal Oblišar, Dalibor Balian, Gregor Skoberne, Mirzeta Memišević Hodžić, Marjana Westergren, Stephen Cavers, Annika Perry, Santiago C. González‐Martínez, Tanja Pyhäjärvi, Ivan Scotti, Giovanni G. Vendramin, Andrea Piotti

**Affiliations:** ^1^ Institute of Biosciences and BioResources (IBBR), National Research Council (CNR) Sesto Fiorentino Italy; ^2^ Department of Forest Sciences University of Helsinki Helsinki Finland; ^3^ Instituto de Ciencias Forestales (ICIFOR‐INIA), Consejo Superior de Investigaciones Cientificas (CSIC) Madrid Spain; ^4^ National Research Institute for Agriculture, Food and the Environment (INRAE), University of Bordeaux, BIOGECO Cestas France; ^5^ National Research Institute for Agriculture, Food and the Environment (INRAE), URFM, Ecology of Mediterranean Forests Avignon France

**Keywords:** forest tree species, genetic conservation units, genetic diversity, genetic monitoring, single primer enrichment technology, targeted genotyping

## Abstract

Genetic diversity is a critical but often overlooked component of biological diversity. The European H2020 FORGENIUS project is aimed at increasing the quality and quantity of genetic data to start monitoring the European network of forest Genetic Conservation Units (GCUs). A first step in this direction was developing standardised genomic resources for 23 forest tree species, spanning from rare and scattered (e.g., *Abies nebrodensis* and *Torminalis glaberrima*) to widespread and stand‐forming, economically relevant ones (e.g., *Fagus sylvatica* , *Picea abies* and *Pinus sylvestris* ). Here, we describe the development and application of targeted genotyping tools, primarily based on Single Primer Enrichment Technology (SPET), along with existing SNP arrays for the selected species. The SPET panels developed in FORGENIUS were designed to capture ⁓10,000 loci per species, balancing species‐specific and randomly distributed regions to ensure broad genome coverage and minimise ascertainment bias. Across 7220 genotyped trees, we identified over 1.8 million single nucleotide polymorphisms (SNPs) covering approximately 50 Mb of DNA sequence. SPET panels demonstrated high genotyping efficiency and cross‐species transferability, especially within genera such as *Quercus* and *Abies*. They represent a cost‐effective, flexible, and scalable solution for population‐level genetic assessments across diverse taxa, enabling standardised, genome‐wide characterisation of the GCU network. These resources not only promote the establishment of genetic monitoring, support genetically informed conservation strategies and improve our understanding of adaptive responses in European forests, but also enhance species delimitation and hybrid detection, and enable the characterisation of phylogenetically related but previously underexplored species.

## 1 Introduction

Despite being a key component of biodiversity, genetic diversity has been long overlooked in international conservation programs (Laikre et al. 2020) until very recent initiatives (Hoban et al. 2025). Up to 16% of global genetic diversity is expected to go extinct by the end of the 21st century due to habitat loss (Exposito‐Alonso et al. 2022) and proper genomic tools are crucial to track its future changes (Pearman et al. 2024; Shaw et al. 2025). Genetic monitoring must rely on population surveys or inventories that can be repeated through time (Schwartz et al. 2007). To this aim, an asset for European forests is the continent‐wide network of in situ Genetic Conservation Units (GCUs) coordinated by the European Forest Genetic Resources Programme (EUFORGEN) since 1994 (Lefèvre et al. 2013) and accessible through the EUFGIS information system (http://portal.eufgis.org). The GCU network is designed to represent forest tree stands that are adapted to unique sets of environmental conditions and are thus expected to have distinct genetic, phenotypic, and/or ecological characteristics.

Decisions on the inclusion of GCUs into the EUFORGEN collection have mostly been based on non‐standardised local observations, rather than on quantitative scientific assessments (de Vries et al. 2015). For instance, genetic information is lacking in the EUFGIS information system, as well as information about structural, physiological and climatic characteristics of the forest stands in the GCUs. In addition, the proxy used to identify differently adapted GCUs (i.e., climatic zonation) omits many other potential drivers of natural selection and is far from optimal. The urgent need for a deep, functional characterisation of GCUs requires information about adaptive variation at the molecular and phenotypic levels (EUFORGEN 2021), and its integration with environmental data. Although a few, recently published studies include both a range‐ and genome‐wide characterisation of Europe's most iconic tree species (e.g., Bruxaux et al. 2024; Milesi et al. 2024; Theraroz et al. 2024; Zhou et al. 2024), much of the genetic information on the few genetically characterised European forest tree species is still based on a limited number of neutral molecular markers (Aravanopoulos et al. 2015) that does not adequately describe their genetic diversity, in general, and, in particular, their adaptive potential. Since the advent of high‐throughput sequencing techniques, however, it is possible to apply the same genotyping standard—sequencing itself or sequence‐based genotyping—to all species, making genomic information homogeneous in kind (if not in completeness). Indeed, one of the overarching goals of the European H2020 project FORGENIUS (http://www.forgenius.eu) is to address the lack of knowledge on the European GCU network and provide a common baseline for monitoring the levels of neutral and adaptive genetic variation in European forests through time.

Several techniques based on next‐generation sequencing, including RAD‐Seq, exome capture, Pool‐Seq, and whole‐genome resequencing (WGS), as well as DNA arrays, are widely used for rapid and cost‐effective genomic characterisation of natural resources, each offering specific strengths and limitations depending on research goals, sample sizes, and available resources. SNP arrays offer low per‐sample cost and high throughput but rely on predefined variant sets that may introduce ascertainment bias and limit their transferability across populations or species (Plomion et al. 2016). Conversely, WGS provides the most comprehensive assessment of genomic variation but remains substantially more expensive for large‐scale population studies and is still prohibitive for species with extremely large genomes, such as many conifers, due to the dramatically increased sequencing and computational demands despite declining sequencing costs (Benjelloun et al. 2019). The targeted genotyping system based on Single Primer Enrichment Technology (SPET, Scaglione et al. 2019) is an attractive alternative that is becoming increasingly popular. SPET uses a single primer to selectively enrich specific regions of the genome, enabling targeted genotyping with reduced sequencing costs. This approach efficiently targets single nucleotide polymorphisms (SNPs) of interest while also yielding hundreds of thousands of untargeted variable sites (referred to as target and *de novo* SNPs, respectively). The sequencing read length can be adjusted based on the need to detect unknown (*de novo*) SNPs or to focus on cost‐effective target genotyping. Compared to SNP arrays, SPET enables the targeting of randomly distributed genomic regions, which allows markers to be generated with minimal ascertainment bias and improves applicability across populations and species. Compared to WGS, SPET significantly reduces sequencing effort while still generating genome‐wide information at sufficient density for population genomics and conservation applications. This balance between flexibility, resolution, and cost‐efficiency is one of the main reasons for its increasing use in ecological and forest genomics studies (Budde et al. 2024; Olsson et al. 2023). SPET panels can be easily transferred to other populations or even species and applied across different laboratories, facilitating the integration of data from multiple sources. The high reproducibility of SPET data (Scaglione et al. 2019) makes it an excellent tool for population genomic analysis of natural populations and genetic monitoring, especially while WGS remains impractical for large‐sample studies or for species with very large genomes.

Here, we describe the genomic resources developed and data produced for 23 forest tree species within the European H2020 FORGENIUS project (https://www.forgenius.eu/). Five species were genotyped using existing, well‐established tools based on array technology ( *Pinus pinaster* , *Pinus pinea* , *Pinus sylvestris* , *Populus nigra* and *Populus alba* ), while for all the other species we developed new SPET panels. These panels were designed to target approximately 10,000 genomic loci, uniformly distributed across the genome, combining both randomly selected regions and species‐specific regions of interest. Using SPET panels and SNP array chips, we identified a total of 1,850,982 SNPs across 7220 trees, covering about 50 Mb of genomic sequence. We describe the development of these genomic resources, from the design of SPET panels to data production, highlighting the different strategies adopted to obtain equally high‐quality markers despite species‐to‐species variation in properties, such as presence/absence of a reference genome or proportion of repeated elements in the genome (which varies considerably between angiosperms and gymnosperms). We present overall results of genetic diversity and structure at the species level, emphasising the importance of including a fraction of randomly selected loci in the set of target genomic regions, to ensure that estimates are unaffected by ascertainment bias. Finally, we discuss the relevance of the FORGENIUS genomic resources for the genomic characterisation of the European network of GCUs, as well as their broader applications, ranging from genetic monitoring and the assessment of genomic vulnerability to the study of within‐population dynamics (gene flow, hybridization, sexual vs. vegetative reproduction, etc.) and the reconstruction of past evolutionary history.

## 2 Materials and Methods

### 2.1 Genomic Characterisation of Forest Genetic Conservation Units (GCUs)

**FIGURE 1:**
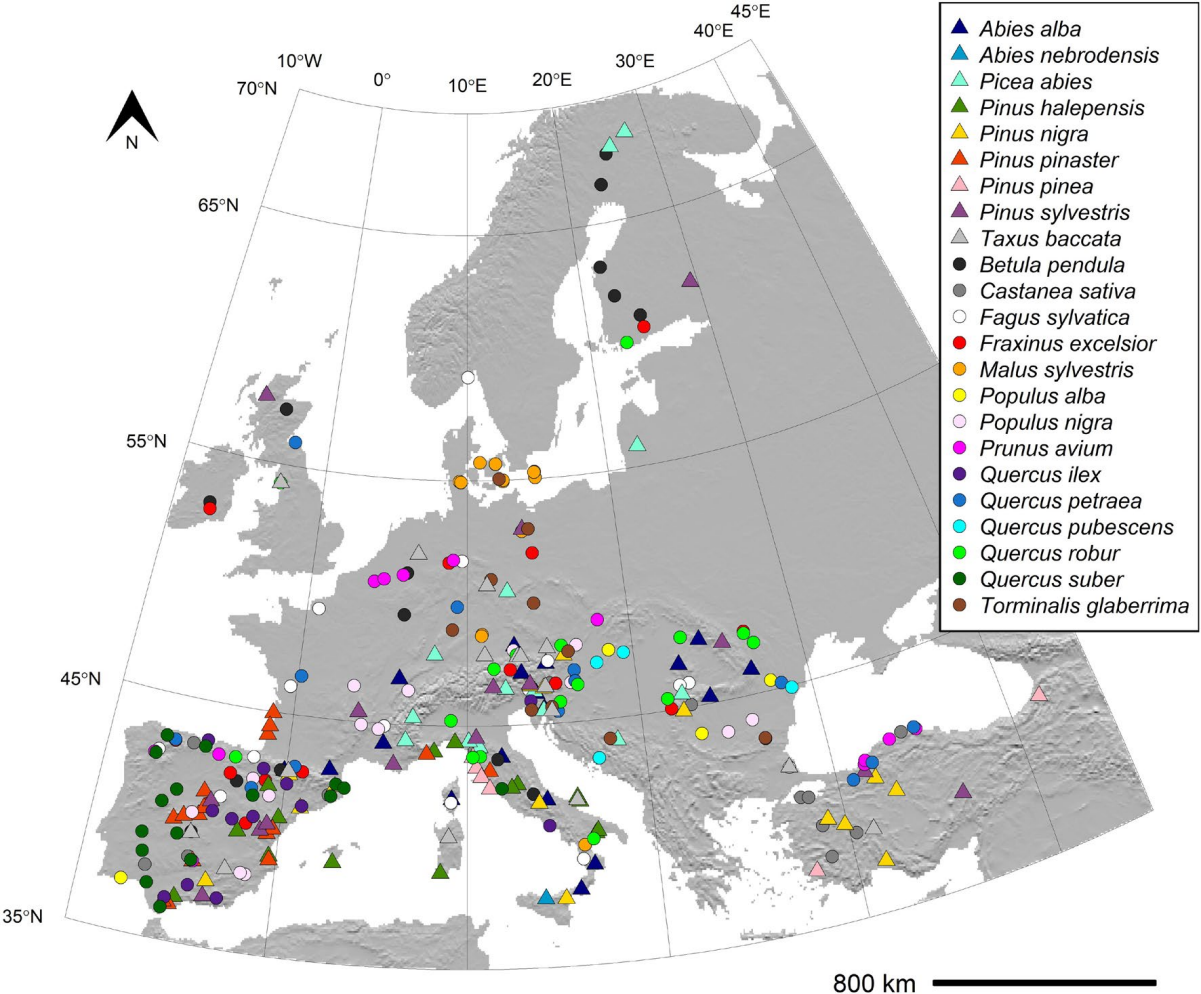
Distribution of the 301 GCUs of 23 forest tree species sampled for genomic characterisation (details in Table S1). Different symbols refer to conifers (triangles) and broad‐leaved species (circles).

Twenty‐three forest tree species were qualitatively selected within FORGENIUS to provide a detailed and in‐depth characterisation of a representative subset of the GCU network, as they globally occur in ⁓80% of GCUs. The selected species range from some of the most widespread ecologically and economically relevant in Europe (e.g., *Fagus sylvatica* , *Pinus sylvestris* , *Picea abies* , *Quercus petraea* ) to rare species, such as *Abies nebrodensis*, or scattered species, such as *Malus sylvestris* and *Torminalis glaberrima* (formerly *Sorbus torminalis* ). They cover a suite of environmental requirements, are present in most European eco‐regions, and correspond to multiple societal demands (Figure 1).

Within each species, GCUs were selected to cover all environmental zones in which they occur (i.e., at least one GCU per environmental zones, as defined by de Vries et al. 2015), as well as to include ecologically and geographically marginal populations. The number of GCUs characterised per species spans from one (the only existing population of *A. nebrodensis* ) to 18 ( *Abies alba* ) and is approximately proportional to the total number of GCUs for the focal species, totalling 301 GCUs for which genomic data were generated (Figure 1, Table S1).

### 2.2 Single Primer Enrichment Technology (SPET) Panels Design

Species‐specific SPET probes were custom designed for fourteen species on the latest version of their reference genome, if available, or on the transcriptome sequence. Details on the reference sequences used for probe design are provided in Table 1.

**TABLE 1:** List of reference genomes or transcriptomes used for probe design and for data analysis.

Species	Reference for probe design	Reference for data analysis
*Abies alba*	*Abies alba* Abal.1_1 genome (Mosca et al. 2019)	*Abies alba* Abal.1_1 genome (Mosca et al. 2019)
*Betula pendula*	*Betula pendula* Bpev01 genome (Salojärvi et al. 2017)	*Betula pendula* Bpev01 genome (Salojärvi et al. 2017)
*Castanea sativa*	*Castanea sativa* transcriptome (Acquadro et al. 2019)	*Castanea mollissima* Mahogany genome (http://phytozome.jgi.doe.gov/info/CmollissimaMahoganyHAP1_v1_1)
*Fagus sylvatica*	*Fagus sylvatica* v2 genome (Mishra et al. 2022)	*Fagus sylvatica* v2 genome (Mishra et al. 2022)
*Fraxinus excelsior*	*Fraxinus excelsior* FRAX‐001 genome (PRJNA713541)	*Fraxinus excelsior* FRAX‐001 genome (PRJNA713541)
*Malus sylvestris*	*Malus sylvestris* haploid_v2 genome (PRJNA599188)	*Malus sylvestris* haploid_v2 genome (PRJNA599188)
*Picea abies*	*Picea abies* 1.0 genome (Nystedt et al. 2013)	*Picea abies* 1.0 genome (Nystedt et al. 2013)
*Pinus halepensis*	*Pinus halepensis* transcriptome (Pinosio et al. 2014)	*Pinus tabuliformis* genome v1.0 (Kesälahti et al. 2025)
*Pinus nigra*	*Pinus nigra* transcriptome (Olsson et al. 2020)	*Pinus tabuliformis* genome v1.0 (Kesälahti et al. 2025)
*Prunus avium*	*Prunus avium* Tieton_v2.0 genome (Wang et al. 2020)	*Prunus avium* Tieton_v2.0 genome (Wang et al. 2020)
*Quercus ilex*	*Quercus ilex* MR‐2023 genome (Rey et al. 2023)	*Quercus ilex* MR‐2023 genome (Rey et al. 2023)
*Quercus robur*	*Quercus robur* PM1N genome (Plomion et al. 2018)	*Quercus robur* PM1N genome (Plomion et al. 2018)
*Taxus baccata*	*Taxus baccata* transcriptome (Olsson et al. 2018)	*Taxus chinensis* genome (Xiong et al. 2021)
*Torminalis glaberrima*	*Torminalis glaberrima* transcriptome (Pinosio et al. 2025)	*Sorbus pohuashanensis* genome (Zhao et al. 2022)

For all species genotyped by SPET, at least half of the probes were designed to capture random sites of the genome/transcriptome to obtain a set of SNPs without ascertainment bias. The number of random and target sites for each species is reported in Table S2. In the species for which molecular markers were already available, probes targeting known SNPs were also included. For each species, excluding *Quercus robur* and *F. sylvatica* , the corresponding reference genome or transcriptome, along with a BED file containing the selected target SNPs and random sites, were submitted to Tecan Genomics (Männedorf, Zurich, Switzerland) for probe design. For *Q. robur* and *F. sylvatica* , probes targeting both random sites and SNPs were selected from two pre‐existing 90 K SPET panels (Budde et al. 2024; Tost et al. 2025). A total of four distinct SPET probe panels were developed: (1) a species‐specific panel including 10,000 probes for *A. alba* (FORGENIUS‐Aalb10K), (2) a species‐specific panel including 10,000 probes for *Pinus halepensis* (FORGENIUS‐Phal10K), (3) a multi‐species panel comprising 60,000 probes (FORGENIUS‐MultiSp60K) with 10,000 probes designed for each of the six included species (i.e., *Betula pendula* , *Fraxinus excelsior* , *M. sylvestris* , *Pinus nigra* , *Prunus avium* , and *Taxus baccata* ), and (4) a multi‐species panel comprising 53,500 probes (FORGENIUS‐MultiSp50K), including 10,000 probes for *Castanea sativa* , *F. sylvatica* , *P. abies* , *Q. robur* , and *T. glaberrima* , along with 3500 probes for *Quercus ilex* . These FORGENIUS SPET panels are available at IGATech (https://igatechnology.com/sequencing‐services/targeted‐genotyping/). Table S3 provides detailed information on each panel, including probe sequences, target site positions, and site type (i.e., target SNP or random site).

### 2.3 Selection of Random Target Sites for Probe Design on Reference Genomes and Transcriptomes

Whenever a reference genome was available, probes targeting random sites of the genome were chosen on non‐repetitive regions using a k‐mer approach. To this aim, the Tallymer tool (Kurtz et al. 2008) included in the GenomeTools suite version 1.4.1 (Gremme et al. 2013) was run to estimate the mappability along the genome by calculating the number of occurrences of each genomic 20‐mer. First, the subroutine suffixerator was used to construct the enhanced suffix array of the reference genome with options ‐dna ‐pl ‐tis ‐suf ‐lcp ‐v ‐parts 16 to (i) specify DNA input, (ii) generate the prefix table (‐pl), suffix array (‐suf), and longest common prefix table (‐lcp), (iii) store intermediate tables on disk (‐tis) to reduce memory usage, (iv) enable verbose output (‐v), and (v) split the computation into 16 parts (‐parts 16) to improve performance on large genomes. The resulting suffix array was then used by the Tallymer subroutine mkindex to build a 20‐mer index with ‐mersize 20 to balance uniqueness and computational efficiency, ‐minocc 1 to retain all k‐mers occurring at least once, and ‐counts to store exact k‐mer frequencies for quantifying genomic repetitiveness. This index was subsequently queried using tallymer search to count the occurrences of each 20‐mer across the genome, providing a quantitative estimate of local repetitiveness. Thus, random positions were selected in regions at least 200 bp long and characterised by an average 20‐mer value lower than 2.

When a reference genome was unavailable and a transcriptome was used instead, the genome of a related species was used to identify regions with high mappability that were also contiguous between the transcriptome and genome. This approach ensured that probes were not designed across exon‐intron junctions or within repetitive sequences. To this aim, the *wgsim* utility included in the samtools v1.7 package (Danecek et al. 2021) was used to simulate 4 million paired‐end 150 bp reads from each reference transcriptome. We simulated 4 million paired‐end reads of 150 bp each (‐1 150 ‐2 150 ‐N 4000000) to obtain a sufficient sequencing depth for downstream analyses while keeping the dataset computationally manageable. The mean insert size was set to 400 bp with a standard deviation of 25 bp (‐d 400 ‐s 25) to mimic typical Illumina paired‐end libraries. All error and mutation rates were set to zero (‐e 0 ‐r 0 ‐R 0 ‐X 0) to generate perfect reads, which allowed us to assess the mapping and coverage along the reference transcriptomes without introducing sequencing artefacts. Simulated reads were aligned to the genome of a related species using the short read aligner BWA‐MEM (Li and Durbin 2009) with default parameters. Uniquely aligned and properly paired reads were selected and used to define the regions of contiguity between the transcriptome and the genome on which random sites were selected.

### 2.4 Selection of Known Target Sites for Probe Design

For four species ( *A. alba* , *P. halepensis* , *Pinus nigra* , and *T. baccata* ), known target SNPs were selected from SPET panels developed within the European H2020 GENTREE project (https://www.gentree‐h2020.eu/) to capture species‐specific regions of interest, also including orthologous genes involved in stress responses and local adaptation. For *B. pendula* , *F. sylvatica* , *P. abies* , and *Q. robur* , known target SNPs were selected from a dataset of genetic polymorphisms previously identified by an exome‐capture sequencing experiment targeting orthologous genes involved in putative functions of interest such as response to stress, immune response, circadian clock, and detection of abiotic stimulus (Milesi et al. 2024). For *Q. robur* , the known target set also included species‐discriminatory markers (Guichoux et al. 2013; Nocchi et al. 2022; Reutimann et al. 2020) and additional candidate genes associated with responses to abiotic stressors (Homolka et al. 2013; Rellstab et al. 2016; Saleh et al. 2022; Trudić et al. 2021), pathogen resistance (Bartholomé et al. 2020), and phenology (Derory et al. 2009). For *F. excelsior* , target SNPs were selected from the multispecies ‘4TREE’ SNP chip (Archambeau et al. 2023; Guilbaud et al. 2020) produced within the framework of the European H2020 B4EST project (https://b4est.eu), comprising SNPs located in candidate genes associated with ash dieback disease resistance, emerald ash borer susceptibility, and self‐incompatibility (Kelly et al. 2019; Sollars et al. 2017; Stocks et al. 2019). For *C. sativa* , known target positions included SNPs for varietal identification in European chestnuts (Nunziata et al. 2020), for species, hybrids, and backcross characterisation (Larue et al. 2021), and associated to chestnut gall wasp resistance (Gaudet et al. 2024). *M. sylvestris* known target positions were selected from a 20 K genotyping array (Bianco et al. 2014), while *P. avium* targets were selected from a 6 K genotyping array (Peace et al. 2012) and from markers related to cherry domestication (Pinosio et al. 2020).

### 2.5 DNA Extraction

For all species, DNA was extracted from 20 to 30 mg of dried leaves/needles per individual using NucleoSpin Plant II kit (Macherey‐Nagel). To reach the DNA quality and quantity required both for analyses by SPET (at least 500 ng of DNA/sample with a fluorimetric concentration of 20–50 ng/μL and with absorbance ratios of A_260_/A_280_ ≥ 1.7 and 1.6 ≤ A_260_/A_230_ ≤ 2.2) and SNP chip arrays (a fluorimetric DNA concentration of at least 30 ng/μl with absorbance ratios of 1.8 ≤ A_260_/A_280_ ≤ 2.0 and A_260_/A_230_ > 1.5), the manufacturer's standard protocol was modified by adding 1 ng/μL Proteinase K to the lysis buffer, increasing the lysis process up to 1 h, and, only for *Quercus* spp., *P. avium* and *B. pendula* , by doubling the number of membrane washing cycles with the washing buffer. The CTAB‐based lysis buffer was used for all species except *A. alba* , *P. halepensis* , *Populus alba* and *Populus nigra* , for which the SDS‐based lysis buffer guaranteed higher quality and concentration of DNA.

### 2.6 SNP Array Genotyping

Five species ( *Pinus pinaster* , *Pinus pinea* , *P. sylvestris* , *Populus nigra* and *P. alba* ) were genotyped at Thermo Fisher (Santa Clara, CA) using two different Axiom arrays developed within the European H2020 B4EST project. Specifically, *P. sylvestris* was genotyped using the PiSy50k Axiom array (Kastally et al. 2022) comprising a total of 47,712 SNPs, while the remaining species were genotyped using the multispecies 4TREE Axiom array (Archambeau et al. 2023; Guilbaud et al. 2020). This array comprises a total of 45,893 SNPs, of which 13,408 are for *Populus trichocarpa* , 13,407 are for *P. pinaster* , 5671 are for *P. pinea* and the remaining 13,407 are for *F. excelsior* . The raw data for all species were obtained from Thermo Fisher and analysed using the Axiom Analysis Suite software v5.2, by applying different sample quality and SNP quality thresholds for the two genera (*Populus*: DQC ≥ 0.82, QC call_rate ≥ 95, cr‐cutoff ≥ 97, fld‐cutoff ≥ 3.6, het‐so‐cutoff ≥ −0.1; *Pinus*: DQC ≥ 0.4, QC call_rate ≥ 85, cr‐cutoff ≥ 85, fld‐cutoff ≥ 3.2, het‐so‐cutoff ≥ −0.3).

### 2.7 Library Preparation, SPET Sequencing and SNP Detection

Genomic DNA was quantified using the Qubit 2.0 Fluorometer (Invitrogen, Carlsbad, CA). Libraries were prepared using the *Allegro Targeted Genotyping* protocol from Tecan Genomics (Männedorf, Zurich, Switzerland), using 100 ng of DNA as input and following the manufacturer's instructions, including a 6 bp Unique Molecular Identifier (UMI) incorporated on each original DNA fragment. Libraries were quantified using the Qubit 2.0 Fluorometer, and their size was checked using the High Sensitivity DNA assay from Bioanalyzer (Agilent technologies, Santa Clara, CA). Sequencing was performed at IGA Technology Services (IGATech, Udine, Italy) facilities using a NovaSeq 6000 System (Illumina, San Diego, CA, USA) in paired‐end mode (2 × 150 bp). BCL files from the instruments were processed using the manufacturer's pipeline software to generate FASTQ sequence files.

Adaptor sequences and low quality 3′ ends were removed from DNA short reads using cutadapt (Martin 2011) and ERNE‐FILTER (del Fabbro et al. 2013), with default parameters. After trimming, reads longer than 50 bp were aligned to the respective reference genomes (Table 1) using the short‐read aligner BWA‐MEM with default parameters (Li and Durbin 2009). After alignment, duplicated sequences were removed using custom Perl script that leverages the UMIs added during library preparation (the script is available at 10.6084/m9.figshare.29560976). For each species, working probes were defined as those achieving a sequencing depth of at least 6× at the target sites in at least 50% of the analysed samples. SNP calling was performed on uniquely aligned reads using the software package GATK version v4.5.0.0 (McKenna et al. 2010). First, *HaplotypeCaller* was run in GVCF mode to call potential variant sites at single‐sample level in the SPET target regions. Then, the joint genotyping on the entire cohort of samples of each species was performed using *GenomicsDBImport* and *GenotypeGVCFs* tools.

### 2.8 SNP Filtering and Population Genetic Statistics

From the gVCFs output by GATK, SNPs and invariant sites were temporarily separated for different filtering workflows. SNPs were isolated and filtered with GATK using *SelectVariants* –select‐type‐to‐include SNP –ignore‐non‐ref‐in‐types and *VariantFiltration* –filter‐expression “QD < 2.0 || MQ < 40.0 || MQRankSum < −12.5”. SNPs resulting from ambiguous mapping between paralogous regions were removed based on elevated heterozygosity and read ratios using the Hdplot method with options Hmax = 0.6, RAFmin = 0.2, RAFmax = 0.8, Dmin = −10, Dmax = 10 (McKinney et al. 2017). Genotype filtering was conducted with bcftools version 1.21 (Danecek et al. 2021). Although raw data are provided, we applied lenient filtering to make the dataset broadly accessible and useful for the various research objectives of FORGENIUS users. In particular, only biallelic SNPs were retained, and only loci where “DP ≥ 6”, “GQ ≥ 20”, at least half the samples had a depth greater than six reads, and the minor allele was covered by at least three reads. For each dataset, samples with over 80% missing data were removed. Invariant sites were isolated from the original gVCF with *bcftools view* “–max‐af 0 ‐V snps, indels, mnps, other” and filtered by “DP ≥ 6” where at least half the samples had a depth greater than six reads. Filtered invariant sites were combined with the respective filtered SNPs to create VCFs labelled version “V0”. Code used for filtering is available at DOI: 10.5281/zenodo.15820966.

The V0 filtered VCFs were used to identify and remove outlier and duplicate samples that may have originated while sampling or from other handling errors. To detect possible outliers, a principal component analysis (PCA) was conducted using the R package *snpR* (Hemstrom and Jones 2023) with default settings. This information was combined with inference of individual admixture coefficients using sparse Non‐Negative Matrix Factorization algorithms implemented in the *sNMF* function from R package *LEA* (Frichot et al. 2014) and interpreted in the light of the species biology known from literature and expert consultation. To explore species relatedness and identify putative duplicates, VCFs were formatted with PLINK version 1.9 (Chang et al. 2015) and the KING toolkit version 2.3.2 (Manichaikul et al. 2010) was run three times using the –related, –kinship, and –duplicates flags. If potential duplicates were likely to be natural clones, they were retained; otherwise, one of each pair was removed to produce V1 VCFs. Outliers that were clearly the result of sample mishandling were also removed from V1 files.

Expected heterozygosity (*H*_S_) and Weir & Cockerham's *F*_ST_ (Weir and Cockerham 1984) were estimated at species level using R package *hierfstat* v0.5‐11 (Goudet 2005). Bootstrapping (1000) was used to obtain a 95% confidence interval for the *F*_ST_ values. Nucleotide diversity was estimated using pixy version 1.2.7.beta1 (Korunes and Samuk 2021) on V1 filtered VCF files.

To identify SNPs that discriminate among *Quercus robur* , *Q. petraea* , and *Q. pubescens*, raw VCF files from individuals of each species were merged using bcftools. SNPs were then extracted and filtered using GATK's SelectVariants and VariantFiltration tools, as previously described. Additional filtering was performed with bcftools to retain only biallelic SNPs with a minimum depth ≥ 6, genotype quality ≥ 20, and present in at least 80% of individuals. A PCA was conducted to confirm species identity and exclude putative hybrids or misassigned individuals. For each species, a filtered VCF including only confirmed individuals was generated, and allele frequencies were computed using vcftools. SNPs showing large allele frequency differences (≥ 0.9) between at least two of the three species were retained as species‐discriminant loci.

## 3 Results

### 3.1 SPET Panels Performance

The average number of sequenced read pairs per sample ranged from about 1.3 million in *P. avium* , *Q. ilex* , and *Quercus suber* to about 3.4 million in *Pinus nigra* (Table 2). The alignment rate to the whole genome, considering only uniquely mapped reads, was highly variable among species, ranging from 32.5% when aligning against the highly fragmented 10 Gb genome of *P. abies* to 91.2% when aligning to the pseudomolecule‐level 800 Mb genome of *F. excelsior* . In each species, the majority of the 10 K designed probes were effective, producing an average sequence coverage of at least 6× at the target site in at least half of the genotyped individuals. However, for a group of related species belonging to the Fagaceae family and genotyped with the FORGENIUS‐MultiSp50K SPET panel ( *C. sativa* , *F. sylvatica* , and *Quercus* spp.), the number of effective probes exceeded 10 K due to the cross‐species transferability of a subset of probes. For each of these related species, Table S4 shows the number of effective probes, categorised according to the species they were originally designed for. The average depth obtained at the target regions captured by the working probes ranged from 18.3× in *A. nebrodensis* to 85.1× in *F. excelsior* . The size of the captured genome (i.e., positions of the genome having a sequence coverage ≥ 6×x in at least half of the genotyped samples) ranged from 1.12 Mb in *P. abies* to 3.78 Mb in *F. excelsior* .

**TABLE 2:** Summary of sequencing metrics for each species included in the study. The table reports the average number of sequenced read pairs per sample, the percentage of uniquely aligned reads, the average insert size, the number of working probes, the mean depth at target regions, and the total megabases (Mb) covered across the target space.

Species	Sequenced read pairs	% aligned reads	Insert size	Nr. of working probes	Depth at target regions	Mb covered
*Abies alba*	2,790,277	87.6	261	9627	39.3	1.94
*Abies nebrodensis*	2,457,400	80.7	255	9291	18.3	1.23
*Betula pendula*	1,937,435	76.0	308	7735	61.3	2.35
*Castanea sativa*	1,349,141	88.0	254	14,004	55.6	3.56
*Fagus sylvatica*	1,193,929	83.1	286	11,446	53.5	3.02
*Fraxinus excelsior*	1,887,624	91.2	331	9624	85.1	3.78
*Malus sylvestris*	1,706,058	86.0	297	9139	62.1	2.89
*Picea abies*	2,733,083	32.5	256	9105	21.2	1.12
*Pinus halepensis*	1,795,163	80.9	341	7594	22.5	1.21
*Pinus nigra*	3,451,786	65.0	272	9380	35.0	1.53
*Prunus avium*	1,283,333	89.7	390	9456	41.0	3.52
*Quercus ilex*	1,290,987	87.7	225	10,631	58.3	2.43
*Quercus petraea*	2,084,643	86.3	277	16,511	60.6	4.39
*Quercus pubescens*	1,627,385	78.9	291	16,207	36.7	3.59
*Quercus robur*	2,803,723	86.3	308	16,657	51.0	4.38
*Quercus suber*	1,264,732	86.4	293	9861	50.3	2.52
*Taxus baccata*	2,281,221	75.0	258	9485	59.7	2.61
*Torminalis glaberrima*	2,617,148	91.9	199	9717	149.9	2.74

**FIGURE 2:**
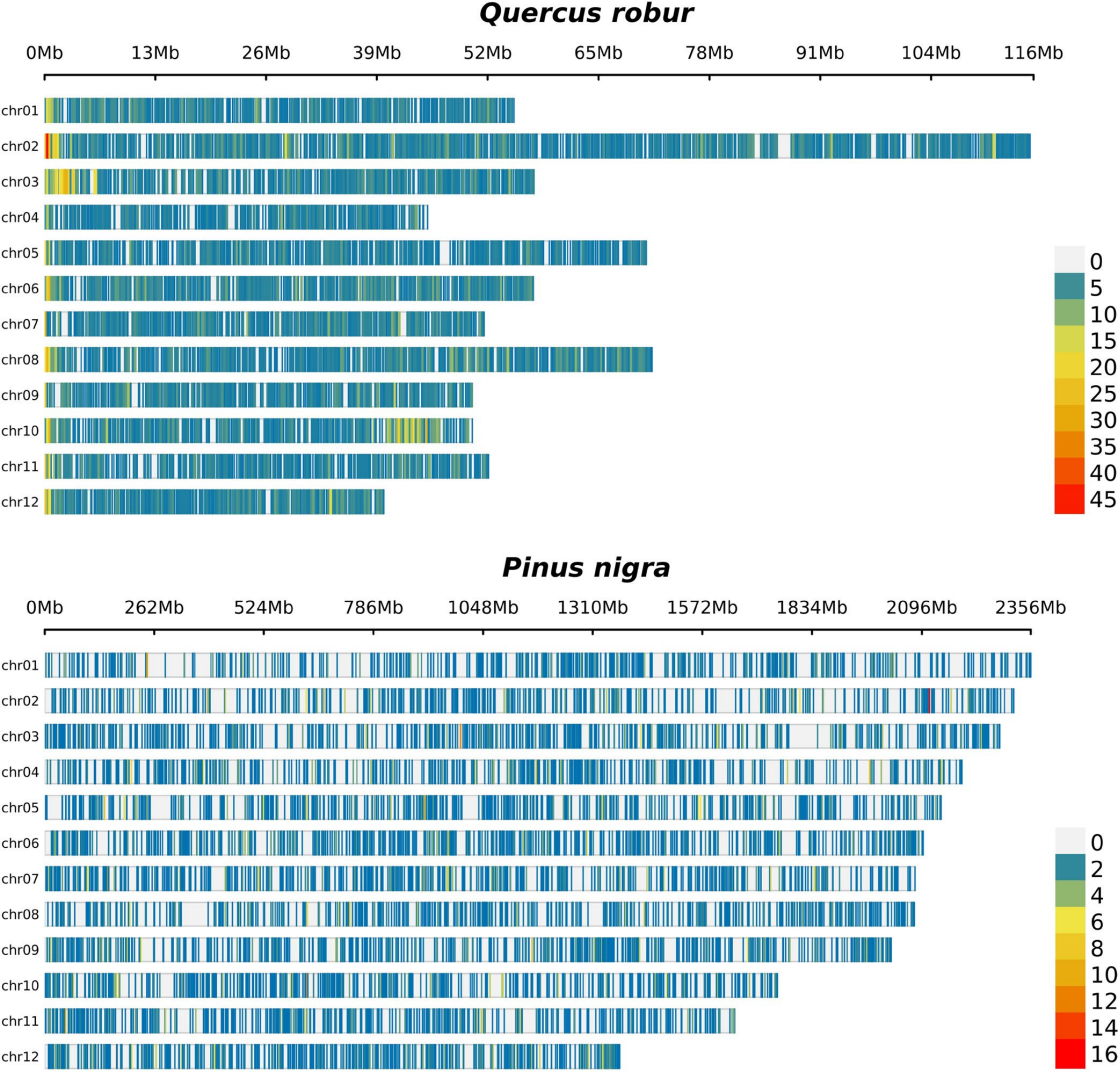
Distribution of *Quercus robur* probes with respect to the *Q. robur* PM1N genome (top) and *Pinus nigra* probes on the *Pinus tabuliformis* genome (bottom), represented as number of probes in 250‐Kb windows.

SPET panels demonstrated high performance when applied to genotype related species. For instance, nearly 9300 of the 10,000 probes designed for *A. alba* successfully genotyped *A. nebrodensis* samples. Similar results were observed in most of the *Quercus* species, where 10,000 probes were designed based on the *Q. robur* genome and 3500 probes based on the *Q. ilex* genome. Of these, 10,700, 11,000, and 4100 probes were effective in genotyping *Q. pubescens*, *Q. petraea* , and *Q. suber* , respectively. Additionally, we tested the SPET panel of *P. halepensis* on a small dataset of *Pinus heldreichii* samples (not included in the 23 species selected in FORGENIUS), and approximately 6700 probes were effective. To visually inspect the genomic distribution of the SPET panels, we plotted the distribution of the working probes in 250 Kb windows for all species with a reference genome at the pseudomolecule level (Figure 2 and Figures S1–S9). Probes were generally well distributed, although consecutive windows without probes were more frequent in species for which probes were designed on the transcriptome ( *C. sativa* and *T. glaberrima* ) and in species with large genomes ( *P. halepensis* , *Pinus nigra* and *T. baccata* ). As an example, Figure 2 shows the distribution of *Q. robur* probes mapped to its own 0.8 Gb genome (top) and *Pinus nigra* probes mapped to the 25.6 Gb *Pinus tabuliformis* genome (bottom). Despite the expected presence of windows without probes in the *P. tabuliformis* genome, the distribution remains relatively uniform across its 12 chromosomes.

### 3.2 SNP Detection and Genomic Characterisation

We genotyped 301 Genetic Conservation Units (GCUs) across 23 forest tree species using either the SPET technology (Scaglione et al. 2019) or the Axiom array‐based genotyping technology (Thermo Fisher, Santa Clara, CA). For the species genotyped using SPET technology, we obtained tens of thousands of SNPs, whereas for those genotyped with Axiom arrays, the number of SNPs was more limited and proportional to the number of markers included in each array (Table 3). The species with the lowest number of detected SNPs was *P. alba* (3031), where most of the markers resulted monomorphic in the 126 genotyped samples, reflecting the use of an array designed for *Populus nigra* . Among the species genotyped by SPET, the lowest number of SNPs was detected in *A. nebrodensis* (16,828), a narrow‐endemic species for which the only 30 existing individuals were genotyped. To assess the reproducibility of SPET genotyping, a total of 48 samples (at least one per species, except for *A. nebrodensis* ) were genotyped twice. On average, 99.1% of genotype calls were concordant between replicates (Figure S10A). Among the discordant calls, the majority (80.2%) were homozygous reference in one replicate and heterozygous in the other, followed by calls that were heterozygous in one replicate and homozygous alternative in the other (16.2%). The least frequent discordances (3.6%) occurred when one replicate was homozygous reference and the other homozygous alternative. Overall, the mean depth at concordant calls was higher compared to discordant ones (Figure S10B). Finally, SNPs in the V1 VCF files were used to calculate, at the species level, expected heterozygosity (*H*_S_) and Weir & Cockerham's *F*_ST_, as well as nucleotide diversity only for SPET‐genotyped species. The results are reported in Table 3.

**TABLE 3:** Number of successfully genotyped samples, name of the genotyping tool, number of SNPs characterised, as well as expected heterozygosity (*H*_S_), nucleotide diversity (*π*), and genetic differentiation (*F*_ST_) per species.

Species	Genotyped samples	Genotyping tool	Number of SNPs	*H*_S_	*π*	*F*_ST_
*Abies alba*	434	FORGENIUS‐Aalb10K	65,629	0.082	0.00246	0.110
*Abies nebrodensis*	24	FORGENIUS‐Aalb10K	16,828	0.243	0.00265	NA
*Betula pendula*	351	FORGENIUS‐MultiSp60K	167,558	0.061	0.00387	0.109
*Castanea sativa*	312	FORGENIUS‐MultiSp50K	64,212	0.150	0.00231	0.236
*Fagus sylvatica*	370	FORGENIUS‐MultiSp50K	113,379	0.098	0.00352	0.116
*Fraxinus excelsior*	331	FORGENIUS‐MultiSp60K	259,272	0.082	0.00538	0.163
*Malus sylvestris*	347	FORGENIUS‐MultiSp60K	114,942	0.115	0.00411	0.087
*Picea abies*	396	FORGENIUS‐MultiSp50K	72,553	0.066	0.00370	0.075
*Pinus halepensis*	344	FORGENIUS‐Phal10K	34,671	0.080	0.00148	0.194
*Pinus nigra*	369	FORGENIUS‐MultiSp60K	93,975	0.061	0.00339	0.078
*Pinus pinaster*	367	4TREE Axiom array	9418	0.266	NA	0.125
*Pinus pinea*	144	4TREE Axiom array	4105	0.171	NA	0.442
*Pinus sylvestris*	368	Pisy50k Axiom array	45,998	0.299	NA	0.058
*Populus alba*	126	4TREE Axiom array	3031	0.095	NA	0.072
*Populus nigra*	329	4TREE Axiom array	10,255	0.207	NA	0.183
*Prunus avium*	262	FORGENIUS‐MultiSp60K	85,249	0.084	0.00181	0.207
*Quercus ilex*	320	FORGENIUS‐MultiSp50K	217,986	0.053	0.00513	0.076
*Quercus petraea*	301	FORGENIUS‐MultiSp50K	244,822	0.050	0.00407	0.048
*Quercus pubescens*	121	FORGENIUS‐MultiSp50K	170,451	0.066	0.00443	0.013
*Quercus robur*	387	FORGENIUS‐MultiSp50K	175,185	0.072	0.00392	0.046
*Quercus suber*	385	FORGENIUS‐MultiSp50K	71,467	0.106	0.00260	0.054
*Taxus baccata*	387	FORGENIUS‐MultiSp60K	52,533	0.176	0.00306	0.196
*Torminalis glaberrima*	244	FORGENIUS‐MultiSp50K	51,350	0.142	0.00237	0.111

For the subset of species within the Fagaceae family where cross‐species transferability of some probes was observed, we assessed whether the use of sequences captured by probes from different species introduced any biases in the estimation of genetic diversity. We calculated observed heterozygosity and minor allele frequency (MAF), stratifying the data according to the origin of the probes. Our analysis revealed no significant differences between the results obtained from species‐specific probes and those captured by probes from other species, suggesting that cross‐species probe transfer did not affect the genetic diversity estimates (Figure S11).

**FIGURE 3:**
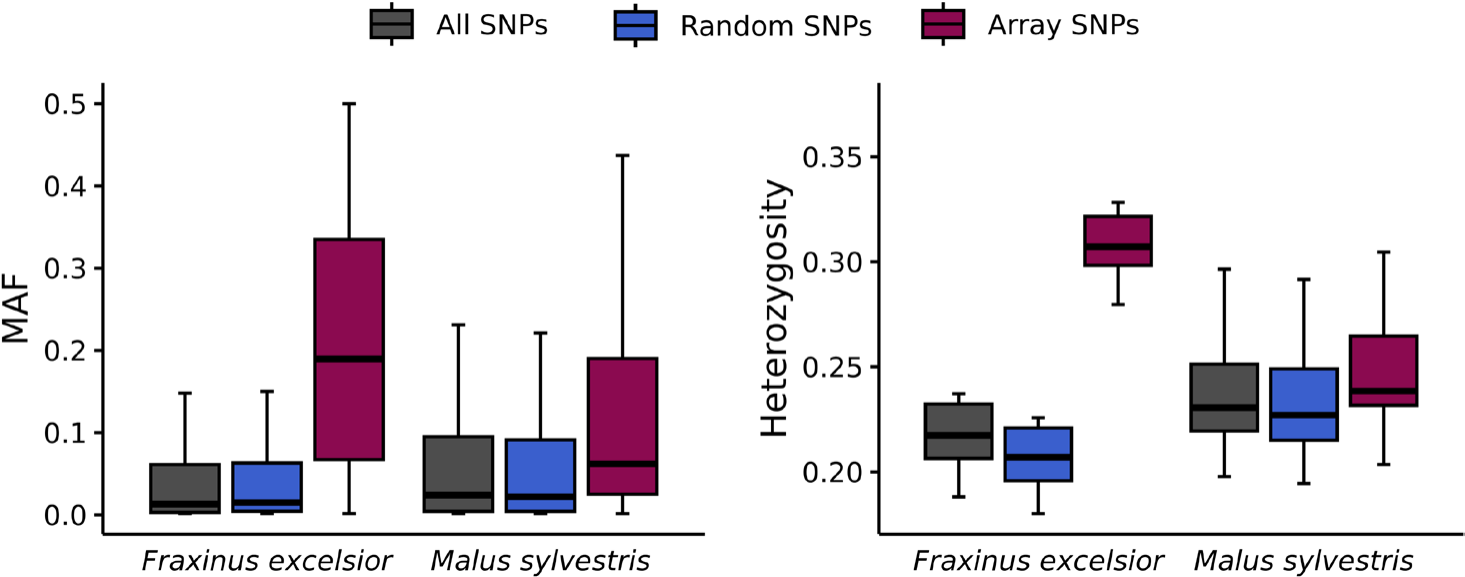
Comparison of genetic diversity estimates based on SPET and SNP array technologies. Boxplots show the distribution of MAF (left panel) and observed heterozygosity (right panel) in *F. excelsior* and *M. sylvestris* , using either the entire dataset (All SNPs), *de novo* SNPs identified in regions captured by random probes (Random SNPs), or SNPs included in the SNP arrays (Array SNPs). Outliers in the boxplots are not displayed to enhance readability.

For *M. sylvestris* and *F. excelsior* a portion of the known target sites included in the SPET panels were derived from two previously developed SNP arrays: an Illumina Infinium array targeting 20 K SNPs for *M. sylvestris* (Bianco et al. 2014) and the multispecies ‘4TREE’ Axiom SNP chip for *F. excelsior* (Guilbaud et al. 2020). This allowed us to compare the genetic diversity estimates obtained for these two species using the two different technologies, either by considering only the subset of SNPs included in the arrays (“array SNPs”) or by analysing *de novo* SNPs identified in regions captured by random probes (“random SNPs”). In both species, we observed a higher MAF and, therefore, observed heterozygosity, in the array SNP set compared to the *de novo* SNP set (Figure 3), as expected due to the preference of SNPs at intermediate frequencies in SNP selection for arrays. This effect was more pronounced in *F. excelsior*, where the differences in the distributions of MAF and heterozygosity were both statistically significant (one‐sided Wilcoxon rank‐sum test *p*‐values 2.2e‐16 and 1.7e‐6, respectively). In contrast, in *M. sylvestris* , only the difference in the MAF distribution was statistically significant (*p*‐value 2.2e‐16). The overestimation of diversity metrics observed in array SNPs determined an underestimation of *F*_ST_ ranging from 5% in *F. excelsior* to 20% in *M. sylvestris* . When the same statistics were calculated for the entire dataset, the estimates were comparable to those obtained from the random probes, indicating that any potential bias introduced by the known target SNPs is mitigated when the data are combined. This is inherently linked to the much larger number of random than target SNPs per probe usually obtained by SPET genotyping (Scaglione et al. 2019). In species for which the reference genome was used for probe design, consistent results were obtained when heterozygosity and *F*_ST_ were calculated separately for target sites and *de novo* SNPs (Table S5). Estimates obtained from the *de novo* datasets were consistent with those obtained from the entire dataset, indicating that any potential bias introduced by using target SNPs for probe design is effectively diluted by the contribution of the more abundant *de novo* SNPs.

The large number of new markers discovered by SPET genotyping enables multi‐purpose applications beyond the original goal of improving the characterisation of GCUs for future genetic monitoring. For instance, the SPET panel developed for *Q. robur* has the potential to capture approximately 200 markers that have been reported to have high discriminatory power among different white oak species (Guichoux et al. 2013; Kremer et al. 2024; Reutimann et al. 2020), listed in Table S6. We also searched for a new set of diagnostic markers by selecting genomic positions where the difference in the frequency of the alternative allele between at least two of the three white oak species was greater than 0.9. Through this approach, we identified a total of 196 new markers with high discriminatory power, as listed in Table S7.

## 4 Discussion

In the framework of the H2020 European project FORGENIUS, new genomic resources were developed and data produced for 23 forest tree species spanning from rare and scattered (e.g., *A. nebrodensis* and *T. glaberrima* ) to widespread, economically relevant ones (e.g., *F. sylvatica* , *P. abies* and *P. sylvestris* ). By designing ad‐hoc SPET panels and employing already available SNP array chips, we identified a total of 1,850,982 SNPs across 7220 trees, covering about 50 Mb of genomic sequence. SPET panels were specifically designed to obtain a wide plateau of genomic information, while minimising potential biases affecting estimates, for the genetic monitoring and the assessment of possible maladaptation under future environmental conditions across the European network of forest Genetic Conservation Units (GCUs). The main features and the potential for a wider usage of the resources presented here are discussed in the following.

### 4.1 Performance and Key Features of FORGENIUS SPET Panels

SPET technology allowed us to generate an extensive set of genetic markers across the analysed species, regardless of genome size or complexity. In fact, even if a higher average coverage was generally obtained in species with smaller and less repetitive genomes, the newly developed SPET designs consistently provided a valuable set of genetic markers well‐distributed across the genomes for all analysed species, including those with larger and more repetitive genomes. For those species for which probes were designed from transcriptome data, it should be noted that the resulting marker set is largely gene‐based and may therefore underestimate genome‐wide neutral diversity relative to markers located in intergenic regions. However, despite this potential limitation, the SPET panels provided the first opportunity to obtain comparable, large‐scale estimates of genetic diversity in these species for which a reference genome is not available.

Notably, the SPET probes demonstrated a high transferability rate among related species, as exemplified by the successful genotyping of *A. nebrodensis* using probes designed for *A. alba* and the effective cross‐species performance observed in the *Quercus* genus. High transferability represents a significant advantage compared to other methods, such as SNP‐genotyping arrays, where inter‐species transferability is often limited (Geraldes et al. 2013; Peace et al. 2012). For example, in the case of *P. alba* , we found a very limited number of polymorphic markers when using the 4TREE array developed for *Populus nigra* . This cross‐species transferability was further supported by results obtained in species of the Fagaceae family genotyped using the Forgenius‐MultiSp50K SPET panel ( *C. sativa* , *F. sylvatica* , and *Quercus* spp.), where the number of effective probes exceeded 10,000 due to the successful amplification of a subset of probes across species. Importantly, the high concordance of genetic diversity estimates obtained using either species‐specific probes or probes originally designed for related species suggests that any ascertainment bias potentially introduced by cross‐species probe application is minimal, if present at all. This versatility suggests that the SPET panels developed in this study could serve as valuable tools for studying genetic diversity in other tree species beyond those included in this work (e.g., the nine Mediterranean firs other than *A. nebrodensis* , Alizoti et al. 2011; the red‐listed *Picea omorika* , Mataruga et al. 2020) Additionally, the ability to employ these panels for comparative analyses across species highlights their utility in evolutionary and ecological studies, offering a scalable and robust approach to genomic characterisation across diverse taxa. Moreover, the availability of multispecies and cross‐transferable SPET panels can help overcome scalability issues often encountered in genotyping projects, particularly when only a limited number of samples need to be genotyped per species. The ability to process multiple species simultaneously in a single run may reduce per‐sample costs and increase genotyping efficiency.

The high transferability of SPET probes among related species highlights the potential of SPET genotyping not only for broad genetic characterisation but also for the identification of species differentiation markers. In the case of white oaks, the SPET panel including *Quercus* species (FORGENIUS‐MultiSp50K) successfully captured approximately 200 markers previously reported as useful for species differentiation (Guichoux et al. 2013; Kremer et al. 2024; Reutimann et al. 2020) and allowed us identify additional 196 markers with high resolution power in distinguishing species. These newly discovered markers, based on range‐wide discovery panels, significantly enhance the ability to differentiate between closely related white oak species, confirming the potential of SPET panels as a powerful tool for species delimitation. Among the wide range of other possible applications, the FORGENIUS SPET panels could also prove useful in reconstructing the origin of material in forest plantations and breeding programs, which is attracting attention for its straightforward applications in predicting the performance of different provenances in relevant climatic set‐ups (e.g., Milesi et al. 2019; Oggioni et al. 2024). The ability to both genotype known markers and discover new ones highlights the effectiveness of SPET genotyping in evolutionary and taxonomic studies (Barchi et al. 2019; Gramazio et al. 2020), providing a scalable and transferable framework for marker discovery across a wide range of species.

In *F. excelsior* and *M. sylvestris* , we were able to evaluate the ability of the SPET designs to capture genetic diversity compared to previously developed SNP arrays showing that SPET panels specifically tailored for these species represent a significant advancement in accurately assessing their range‐wide genetic diversity. Unlike SNP arrays, which are prone to ascertainment bias due to the selection of target polymorphisms from specific populations or studies, the SPET design allowed for the *de novo* detection of SNPs across the entire genome. The differences in diversity and differentiation metrics observed between array SNPs and random SNPs highlight the impact of ascertainment bias in pre‐designed arrays, both in *F. excelsior*, where the disparity was statistically significant for both diversity metrics, and in *M. sylvestris* where the effect was more marked on *F*_ST_ underestimation. Also considering that SPET data, including information on invariant sites, permits estimating an important additional diversity metric, i.e., nucleotide diversity, they overall enable a more comprehensive characterisation of GCUs' genetic composition and more robust conclusions about their evolutionary history and adaptive potential.

### 4.2 Applications of FORGENIUS Genomic Resources to the Conservation of European Forests

The European network of GCUs, established almost 30 years ago and whose georeferenced map was produced by the EUFGIS project (http://www.eufgis.org), has never been systematically characterised for its genetic features. To this aim, it is essential to have tools available for gathering comparable information for a large number of species, which was one of the main goals of the ongoing EU H2020 FORGENIUS project. Genomic resources, both in terms of genotyping tools and data, are in fact basic requirements for starting a genetic monitoring program aimed at assessing GCUs' adaptive capacity, a crucial component of populations' vulnerability (IPCC 2007), through time. Genetic monitoring, i.e., the continuous assessment of populations' genetic diversity, is advocated as a key action to properly conserve and manage natural genetic resources (Pearman et al. 2024) and comply with international commitments for the conservation of biological diversity (e.g., the Convention on Biological Diversity 2022).

The genomic resources presented here proved to be an efficient and affordable tool to characterise the genetic diversity of the European network of GCUs, while providing insights on both future adaptability and past demography, which are key aspects to interpret current forest dynamics and orient conservation strategies. Our parameter estimations at the species level shows the large variety of past demographic histories experienced by FORGENIUS focal species, from the highly panmictic white oaks and *P. sylvestris* (*F*_ST_ ⁓0.05 calculated on populations encompassing a large part of their natural distribution) to highly structured ones reflecting historical fragmentation (e.g., *T. baccata* , Mayol et al. 2015), and/or factors related to limited dispersal and human exploitation (e.g., *C. sativa* , Mattioni et al. 2017; *P. avium* , Pinosio et al. 2020; *P. pinea* , Jaramillo‐Correa et al. 2020). Our estimates of nucleotide diversity and expected heterozygosity are in line with what was found in previous literature, confirming general trends already highlighted in multi‐species studies of forest trees (e.g., the high intraspecific diversity of white oaks as compared to other European anemophilous forest tree species, Milesi et al. 2024), with novel insights on less studied species, such as the relatively high nucleotide diversity of the critically endangered *A. nebrodensis* unique population. Among the FORGENIUS focal species, *M. sylvestris* is the progenitor of a domesticated species, and *P. avium* of widely used cultivars. In both cases, the nucleotide diversity detected in GCUs is relatively similar to estimates obtained studying domesticated varieties (Micheletti et al. 2011; Pinosio et al. 2020). This suggests potentially critical issues in the selection and distribution of GCUs, which would be expected to have higher portion of genetic diversity than related, domesticated varieties. Finally, the presented datasets will generate what, to our knowledge, are the first estimates of nucleotide diversity for species that require special conservation actions (e.g., *A. nebrodensis* , del Valle et al. 2024) and/or will be fundamental elements in future reforestation programs to increase species diversity of European forests ( *T. glaberrima* , Afifi et al. 2025).

Although specific analysis at the GCU level for each species will be the subject of forthcoming studies, for the scientific community interested in using the presented datasets, it is worth noting that the more elaborated VCF files provided (V1) still contain, for instance, hybrid individuals and trees potentially belonging to selected cultivars, because these individuals are considered highly informative on the status of single GCUs as part of the diversity that is preserved within them. These individuals could of course be removed in specific data analyses as needed.

### 4.3 Final Remarks and Future Directions

The FORGENIUS SPET panels and data presented here are intended to describe the status of the European network of forest GCUs, thus representing a first step in developing monitoring programs for the forest genetic resources conserved in this network. Data are available for 23 forest tree species and, considering the high transferability observed in this study at the genus and even family level, the same tools could be applied to a much larger number of species. In addition, our work provides the basis to design SPET panels with promising features on other species as the necessary resources (e.g., full transcriptome and genome reference sequences) become available. For species with limited genome size (ca. 1 Gbp), it will be possible to switch to whole‐genome sequencing soon, although its applicability to thousands of samples is still economically unsuitable. However, even in the case of a strong acceleration on this front, the data produced using the FORGENIUS approach could easily be merged with deeper genomic analyses. For species with extra‐long and complex genomes, such as conifers, the tools presented here could represent a convenient option for characterising the range‐wide genetic diversity of hundreds of populations, which is the sample size required by comprehensive genetic monitoring programs.

The possibility of using a mixture of genetic markers in candidate genes as well as randomly distributed throughout the genome makes SPET data particularly flexible to cover a wide range of research aims, from those related to Gene–Environment Association (GEA) analyses (e.g., genomic offset estimation, Rellstab and Keller 2025) to the assessment of genetic load, mating patterns and demographic analyses, or more conservation‐oriented goals such as those of Spatial Conservation Planning (Vajana et al. 2024). The FORGENIUS SPET panels have been proved ideal tools also to deepen issues related to hybridization and species delimitations, with potentially high accuracy at the whole distribution level, which was problematic when diagnostic markers were developed on geographically limited discovery panels (e.g., Kremer et al. 2024). Finally, we showed how SPET panels transferred to congeneric species could be particularly suitable for comparative genomics analyses, thus providing essential information to forecast forest trees responses to environmental fluctuations (Milesi et al. 2024; Yeaman et al. 2016).

## Author Contributions

Sara Pinosio, Francesca Bagnoli, Camilla Avanzi, Ilaria Spanu, Santiago C. González‐Martínez, Tanja Pyhäjärvi, Ivan Scotti, Giovanni G. Vendramin and Andrea Piotti designed the research. Francesca Bagnoli, Camilla Avanzi, Ilaria Spanu, Maria B. Castellani and Arcangela Frascella performed the lab work. Sara Pinosio, Susan L. McEvoy and Sanna Olsson analysed the data. Andrea Piotti, Tanja Pyhäjärvi, M. Westergren, Ivan Scotti, Giovanni G. Vendramin, Delphine Grivet, Ricardo Alia, Santiago C. González‐Martínez, Stephen Cavers, Berthold Heinze and Camilla Avanzi supervised the selection of samples to create V1 datasets. Francesca Bagnoli, Camilla Avanzi, Ilaria Spanu, Santiago C. González‐Martínez, Maria B. Castellani, Arcangela Frascella, Elia Vajana, Sanna Olsson, Ivan Scotti, Giovanni G. Vendramin, Andrea Piotti and the FORGENIUS Consortium performed the field work. Sara Pinosio, Andrea Piotti, Susan L. McEvoy and Sanna Olsson wrote the manuscript. Francesca Bagnoli, Camilla Avanzi, Elia Vajana, Santiago C. González‐Martínez, Tanja Pyhäjärvi, Ivan Scotti and Giovanni G. Vendramin critical revised the manuscript for important intellectual content. All Authors revised the final version of the manuscript.

## Funding

This work was supported by Horizon 2020 Framework Programme, 862221. ITINERIS project‐Piano Nazionale Ripresa e Resilienza (PNRR), IR0000032.

## Conflicts of Interest

The authors declare no conflicts of interest.

## Supporting information


**Figure S1:** Distribution of *Castanea sativa* probes across the *Castanea mollissima* genome in 250 Kb windows.
**Figure S2:** Distribution of *Fraxinus excelsior* probes across the *Fraxinus excelsior* genome in 250 Kb windows.
**Figure S3:** Distribution of *Fagus sylvatica* probes across the *Fagus sylvatica* genome in 250 Kb windows.
**Figure S4:** Distribution of *Malus sylvestris* probes across the *Malus sylvestris* genome in 250 Kb windows.
**Figure S5:** Distribution of *Pinus halepensis* probes across the *Pinus tabuliformis* genome in 250 Kb windows.
**Figure S6:** Distribution of *Prunus avium* probes across the *Prunus avium* genome in 250 Kb windows.
**Figure S7:** Distribution of probes across the *Quercus ilex* genome in 250 Kb windows.
**Figure S8:** Distribution of *Torminalis glaberrima* probes across the *Sorbus pohuashanensis* genome in 250 Kb windows.
**Figure S9:** Distribution of *Taxus baccata* probes across the *Taxus chinensis* genome in 250 Kb windows.
**Figure S10:** (A) Fraction of concordant genotype calls between replicates. (B) Average sequencing depth at concordant (left) and discordant (right) genotype calls.
**Figure S11:** Comparison of genetic variability estimates obtained by stratifying the data based on the species for which the probes were designed.

**Table S1:** The 301 Genetic Conservation Units genetically characterised within European H2020 FORGENIUS project. Eufgis code, country, environmental zone, geographical location, altitude, and number of genotyped individuals per each GCU, subdivided by species.

**Table S2:** Number of random and target probes included in the SPET panel for each species.

**Table S3:** SPET panels details. The table reports the coordinates, strand, and sequences of the probes included in the four SPET panels, along with the target species, target site, and target type (i.e., known target SNP or random site).

**Table S4:** Number of working probes in the seven species belonging to the Fagaceae family genotyped with the Forgenius‐MultiSp50K SPET panel, categorised by the species on which they were designed.

**Table S5:** Expected heterozygosity (HS) and genetic differentiation (FST) calculated on target SNPs, de novo SNPs and the entire dataset.

**Table S6:** List of species‐differentiation markers for white oaks captured by the *Q. robur* SPET panel, retrieved from the literature (Reutimann et al. 2020; Guichoux et al. 2013; Kremer et al. 2024).

**Table S7:** List of species‐differentiation markers for white oaks de novo detected in this study. For each SNP, the frequency of the alternative allele in *Q. robur* , *Q. petraea* , and Q. pubescens is reported. Two SNPs, highlighted in bold, were previously detected by Guichoux et al. (2013).

## Data Availability

Raw and filtered genotyping data have been uploaded either to the NCBI Sequence Read Archive or the Figshare platform and will be released upon acceptance of the paper. Raw FASTQ data are available on the Sequence Read Archive (SRA) on NCBI under the BioProject IDs PRJNA1183629 (*Abies alba*), PRJNA1187359 (Abies nebrodensis), PRJNA1196663 (Betula pendula), PRJNA1199760 (Castanea sativa), PRJNA1245097 (Fagus sylvatica), PRJNA1198695 (Fraxinus excelsior), PRJNA1202045 (Malus sylvestris), PRJNA1245134 (Picea abies), PRJNA1187483 (Pinus halepensis), PRJNA1191837 (Pinus nigra), PRJNA1214746 (Prunus avium), PRJNA1217833 (Quercus ilex), PRJNA1247253 (Quercus petraea), PRJNA1216914 (Quercus pubescens), PRJNA1247390 (Quercus robur), PRJNA1247367 (Quercus suber), PRJNA1207918 (Taxus baccata), PRJNA1247414 (Torminalis glaberrima). Axiom SNP array raw data for Pinus pinaster, Pinus pinea, Pinus sylvestris, Populus alba, and Populus nigra are available on Figshare at 10.6084/m9.figshare.28787597. Filtered VCF for all species is available on Figshare at 10.6084/m9.figshare.28741400.
