# Supplementary material for: The FORGENIUS Genomic Resources: New Genotyping Tools and Genomic Data for 23 Forest Tree Species and Their Genetic Conservation Units
Source: Mol Ecol Resour. 2026 Mar 3;26(3):e70115. doi: 10.1111/1755-0998.70115 (PMC12954563; doi:10.1111/1755-0998.70115)
Supplement: Supplementary file 1 — Figure S1: Distribution of Castanea sativa probes across the Castanea mollissima genome in 250 Kb windows. Figure S2: Distribution of Fraxinus excelsior probes across the Fraxinus excelsior genome in 250 Kb windows. Figure S3: Distribution of Fagus sylvatica probes across the Fagus sylvatica genome in 250 Kb windows. Figure S4: Distribution of Malus sylvestris probes across the Malus sylvestris genome in 250 Kb windows. Figure S5: Distribution of Pinus halepensis probes across the Pinus tabuliformis genome in 250 Kb windows. Figure S6: Distribution of Prunus avium probes across the Prunus avium genome in 250 Kb windows. Figure S7: Distribution of probes across the Quercus ilex genome in 250 Kb windows. Figure S8: Distribution of Torminalis glaberrima probes across the Sorbus pohuashanensis genome in 250 Kb windows. Figure S9: Distribution of Taxus baccata probes across the Taxus chinensis genome in 250 Kb windows. Figure S10: (A) Fraction of concordant genotype calls between replicates. (B) Average sequencing depth at concordant (left) and discordant (right) genotype calls. Figure S11: Comparison of genetic variability estimates obtained by stratifying the data based on the species for which the probes were designed. [file MEN-26-e70115-s001.docx]

**Supplementary Information for:**

**The FORGENIUS genomic resources: new genotyping tools and genomic data for 23 forest tree species and their Genetic Conservation Units**

Sara Pinosio^1^*, Francesca Bagnoli^1^*, Camilla Avanzi^1^, Maria Beatrice Castellani^1^, Arcangela Frascella^1^, Susan L McEvoy^2^, Sanna Olsson^3^, Ilaria Spanu^1^, Elia Vajana^1^, the FORGENIUS Consortium, Santiago C. González-Martínez^4^, Tanja Pyhäjärvi^2^, Ivan Scotti^5^, Giovanni G Vendramin^1^, Andrea Piotti^1^

**Table of Contents**

| **Figure S1.** Distribution of *Castanea sativa* probes across the *Castanea mollissima* genome in 250 Kb windows. | **Pag. 2** |
| --- | --- |
| **Figure S2.** Distribution of *Fraxinus excelsior* probes across the *Fraxinus excelsior* genome in 250 Kb windows. | **Pag. 2** |
| **Figure S3.** Distribution of *Fagus sylvatica* probes across the *Fagus sylvatica* genome in 250 Kb windows. | **Pag. 3** |
| **Figure S4.** Distribution of *Malus sylvestris* probes across the *Malus sylvestris* genome in 250 Kb windows. | **Pag. 3** |
| **Figure S5.** Distribution of *Pinus halepensis* probes across the *Pinus tabuliformis* genome in 250 Kb windows. | **Pag. 4** |
| **Figure S6.** Distribution of *Prunus avium* probes across the *Prunus avium* genome in 250 Kb windows. | **Pag. 4** |
| **Figure S7.** Distribution of probes across the *Quercus ilex* genome in 250 Kb windows | **Pag. 5** |
| **Figure S8.** Distribution of *Torminalis glaberrima* probes across the *Sorbus pohuashanensis* genome in 250 Kb windows. | **Pag. 5** |
| **Figure S9.** Distribution of *Taxus baccata* probes across the *Taxus chinensis* genome in 250 Kb windows. | **Pag. 6** |
| **Figure S10. A)** Fraction of concordant genotype calls between replicates. **B)** Average sequencing depth at concordant (left) and discordant (right) genotype calls. | **Pag. 6** |
| **Figure S11.** Comparison of genetic variability estimates obtained by stratifying the data based on the species for which the probes were designed. | **Pag. 7** |


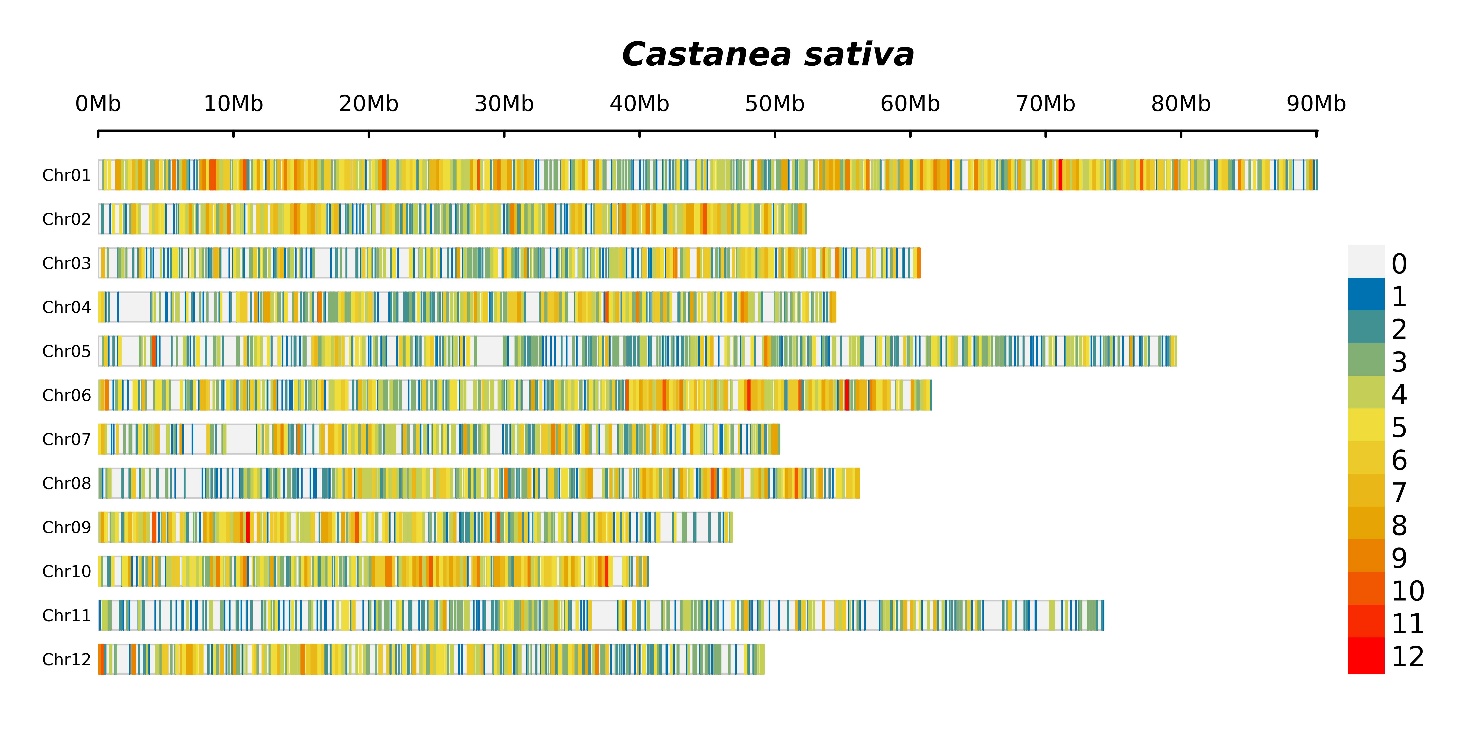


**Figure S1**. Distribution of Castanea sativa probes across the *Castanea mollissima* genome in 250 Kb windows.


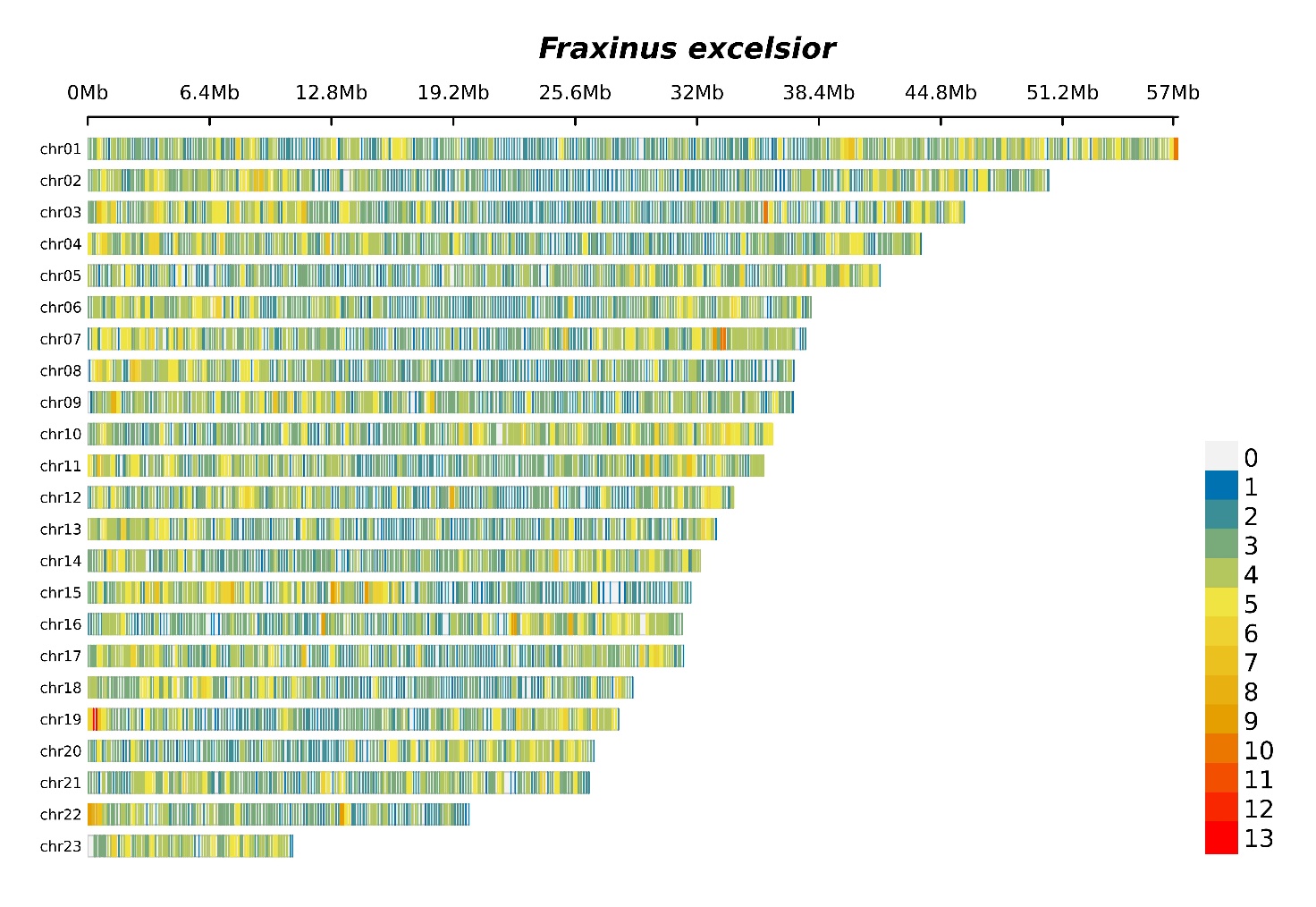


**Figure S2**. Distribution of Fraxinus excelsior probes across the Fraxinus excelsior genome in 250 Kb windows.


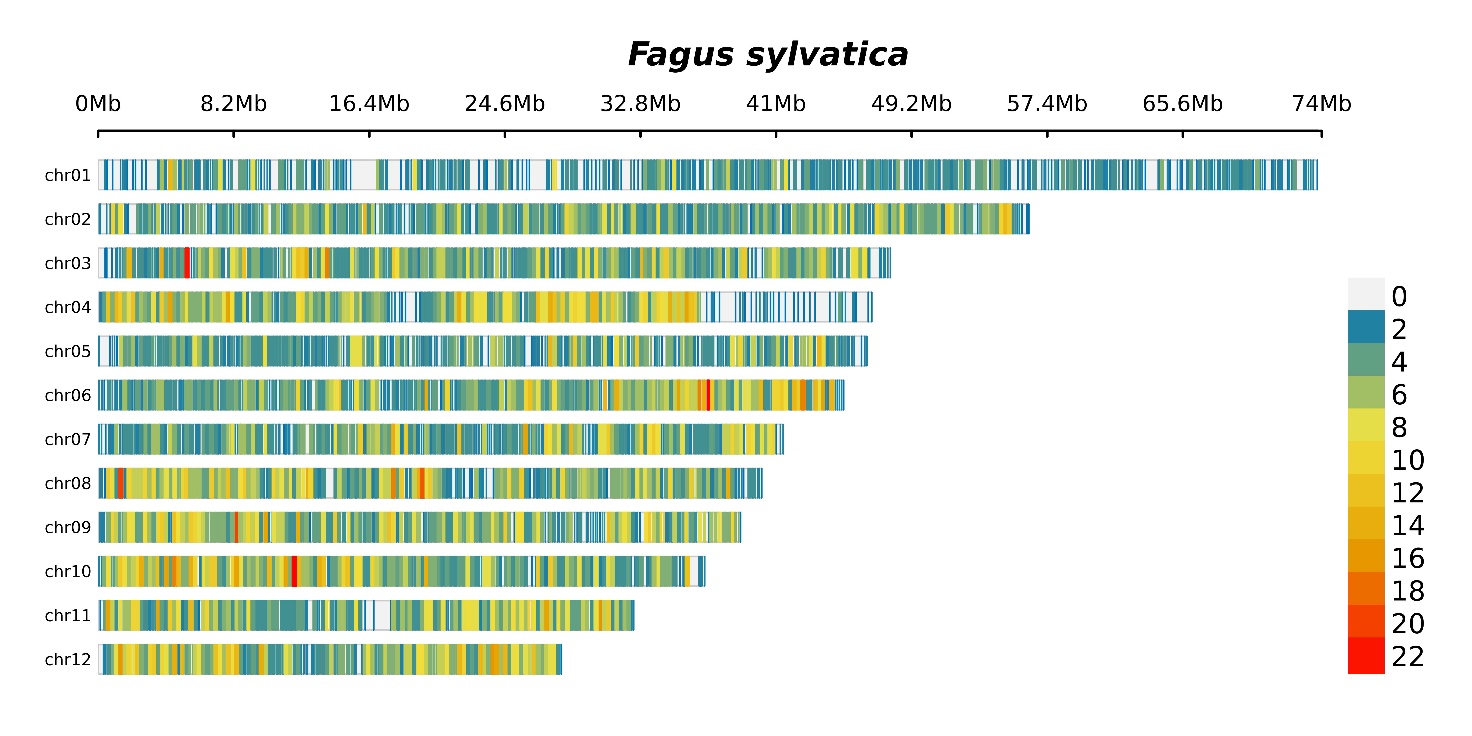
**Figure S3**. Distribution of *Fagus sylvatica* probes across the *Fagus sylvatica* genome in 250 Kb windows.


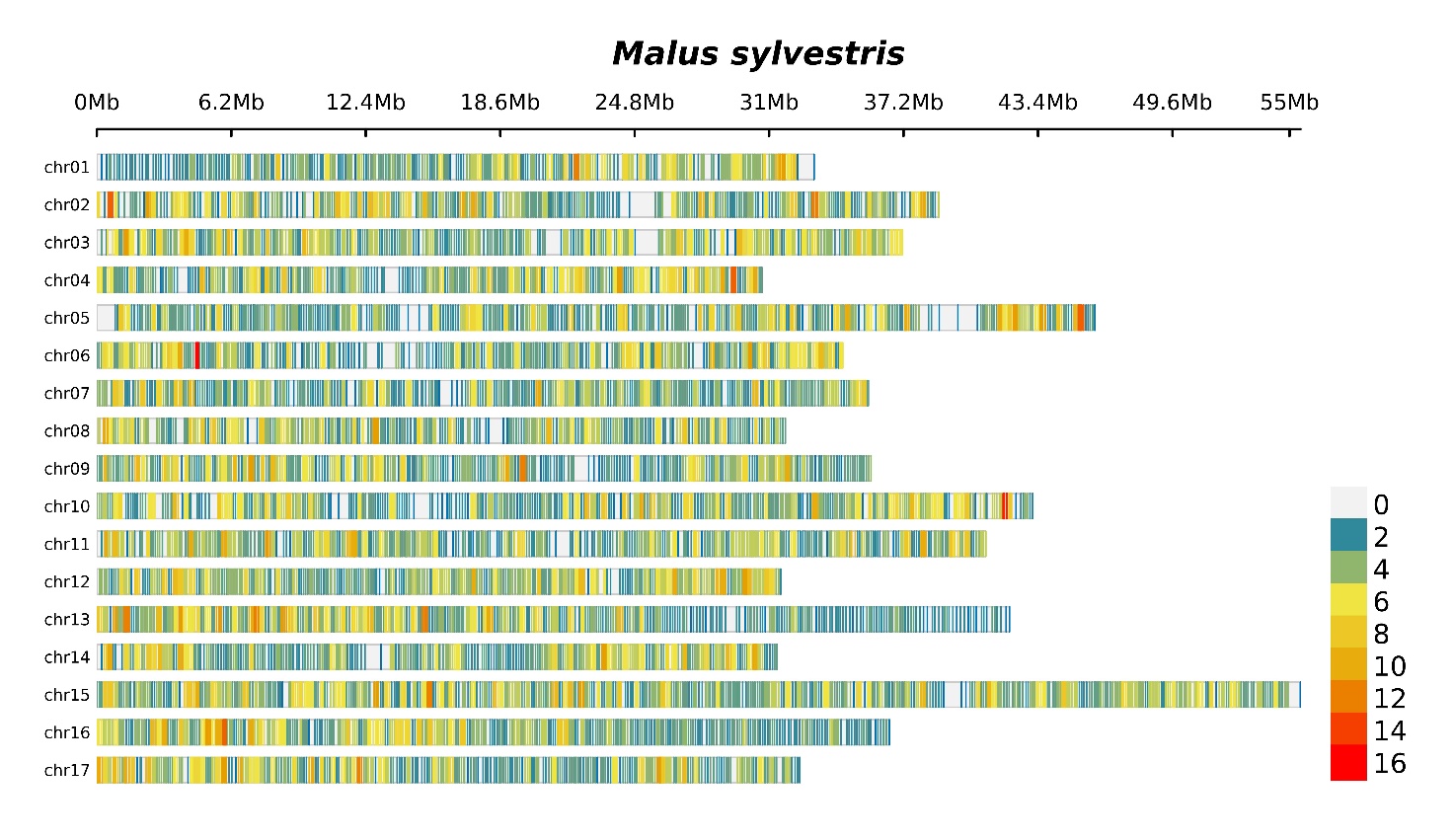
**Figure S4**. Distribution of *Malus sylvestris* probes across the *Malus sylvestris* genome in 250 Kb windows.


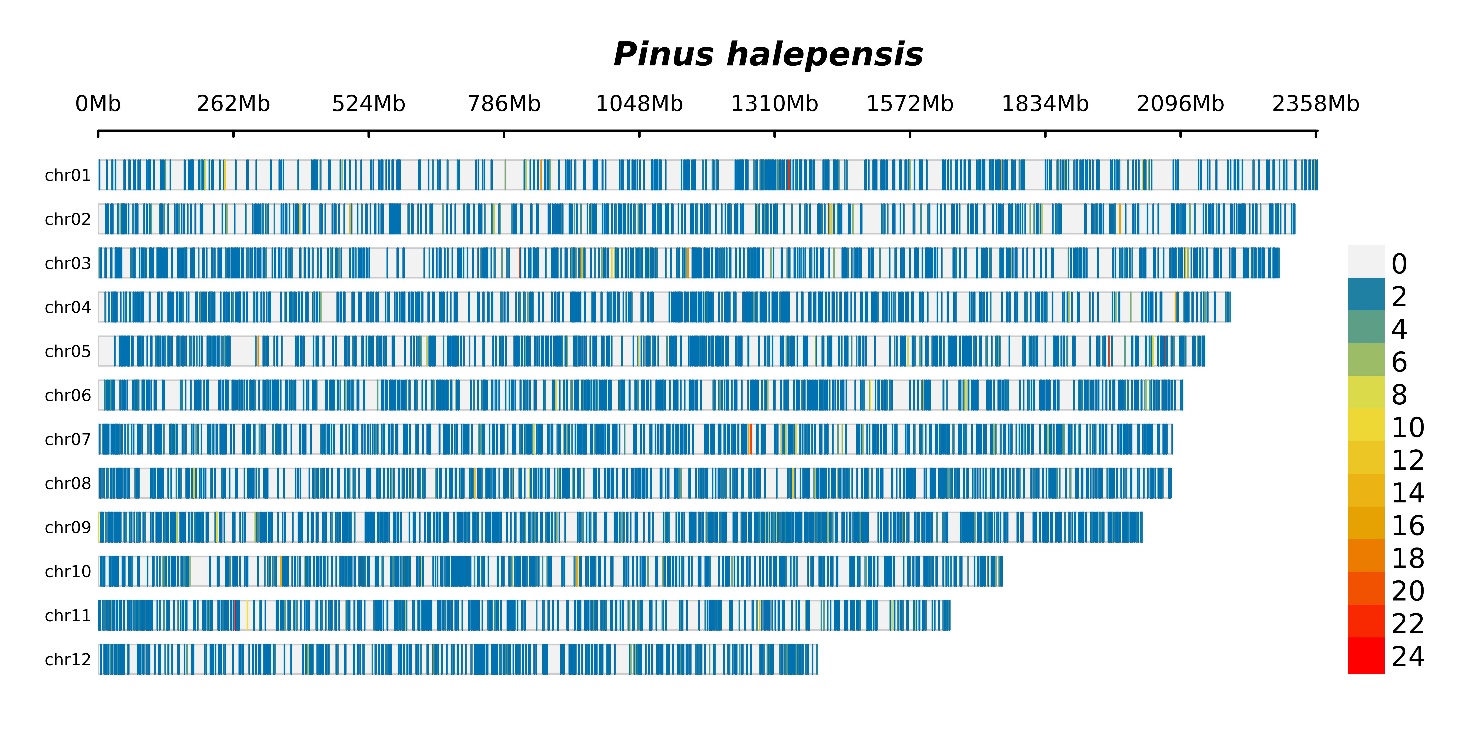
**Figure S5**. Distribution of *Pinus halepensis* probes across the *Pinus tabuliformis* genome in 250 Kb windows.


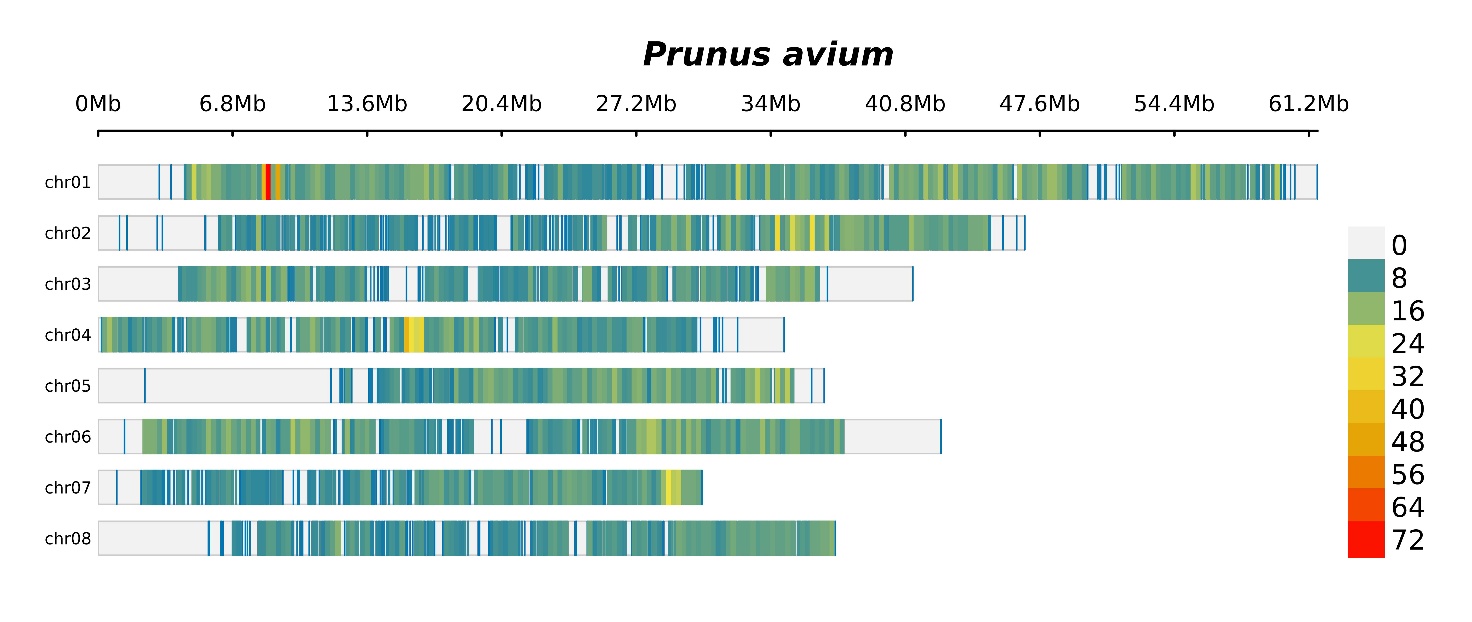
**Figure S6**. Distribution of *Prunus avium* probes across the *Prunus avium* genome in 250 Kb windows.

**
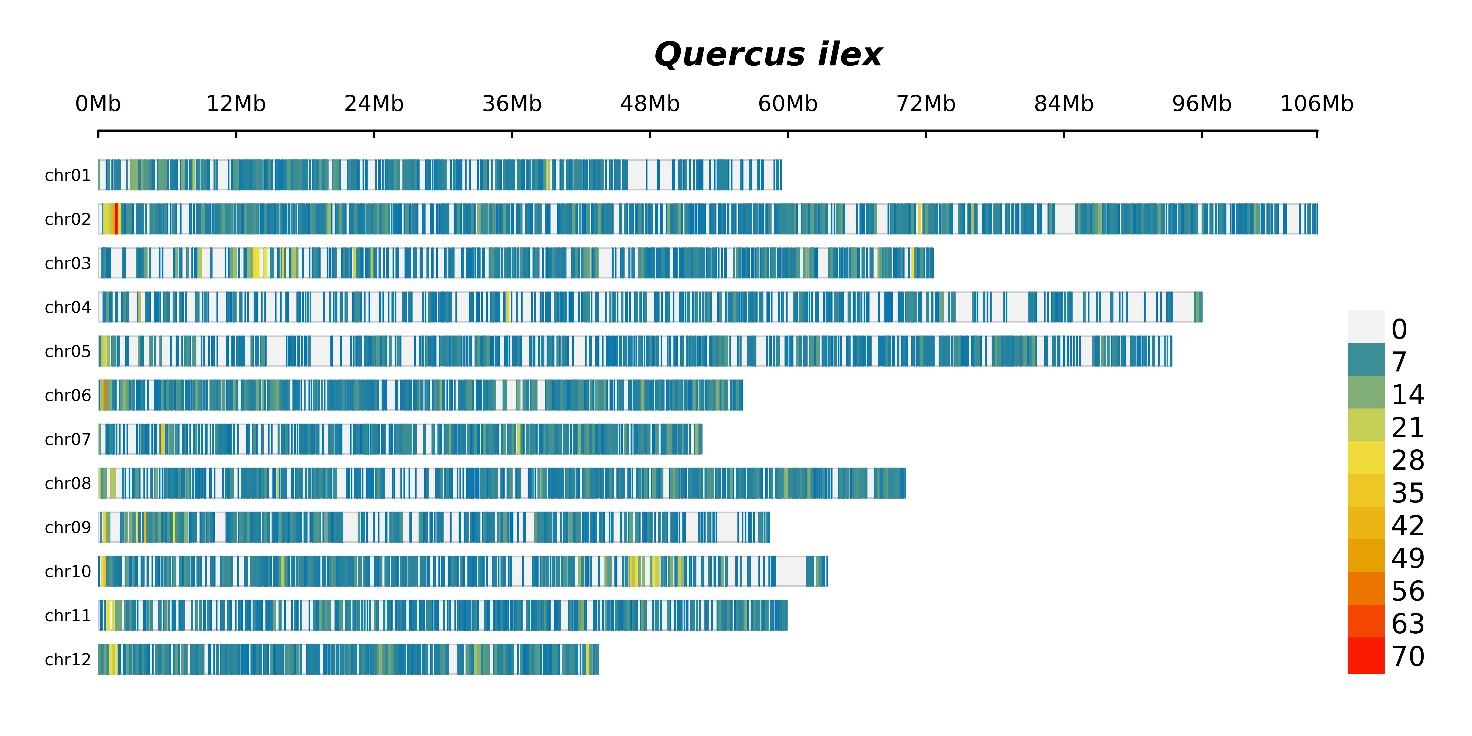
Figure S7**. Distribution of probes across the *Quercus ilex* genome in 250 Kb windows.


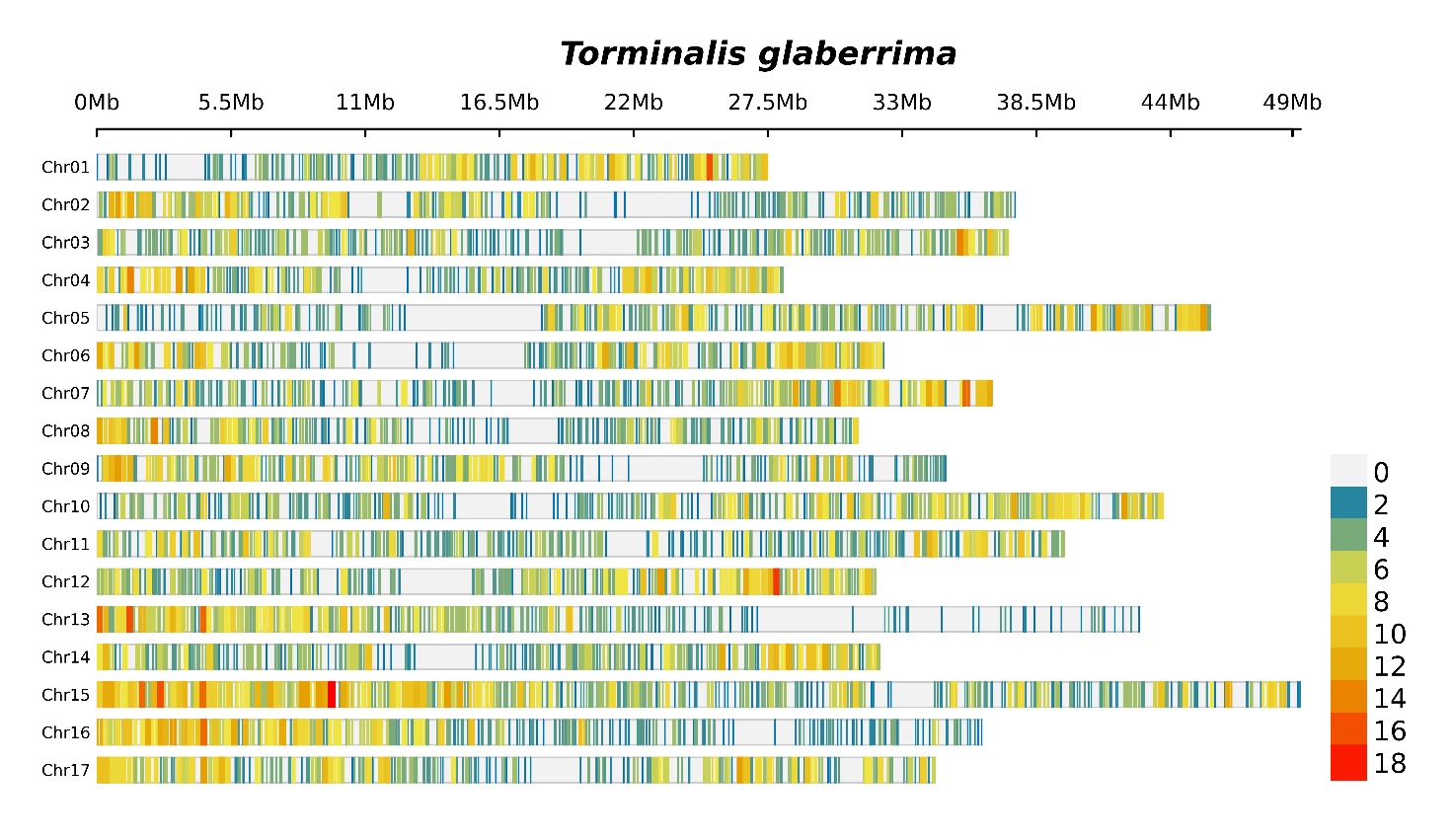
**Figure S8**. Distribution of *Torminalis glaberrima* probes across the *Sorbus pohuashanensis* genome in 250 Kb windows.


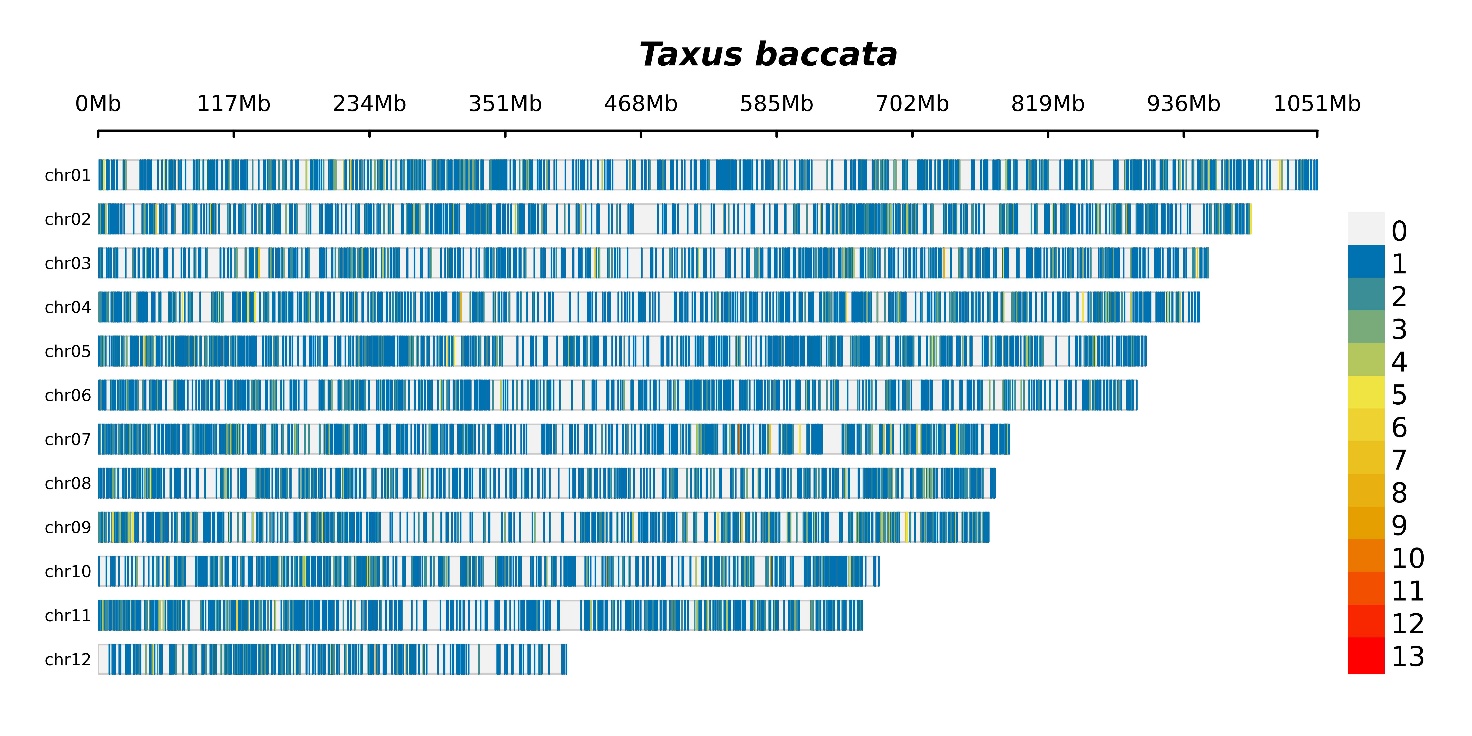
**Figure S9**. Distribution of *Taxus baccata* probes across the *Taxus chinensis* genome in 250 Kb windows.


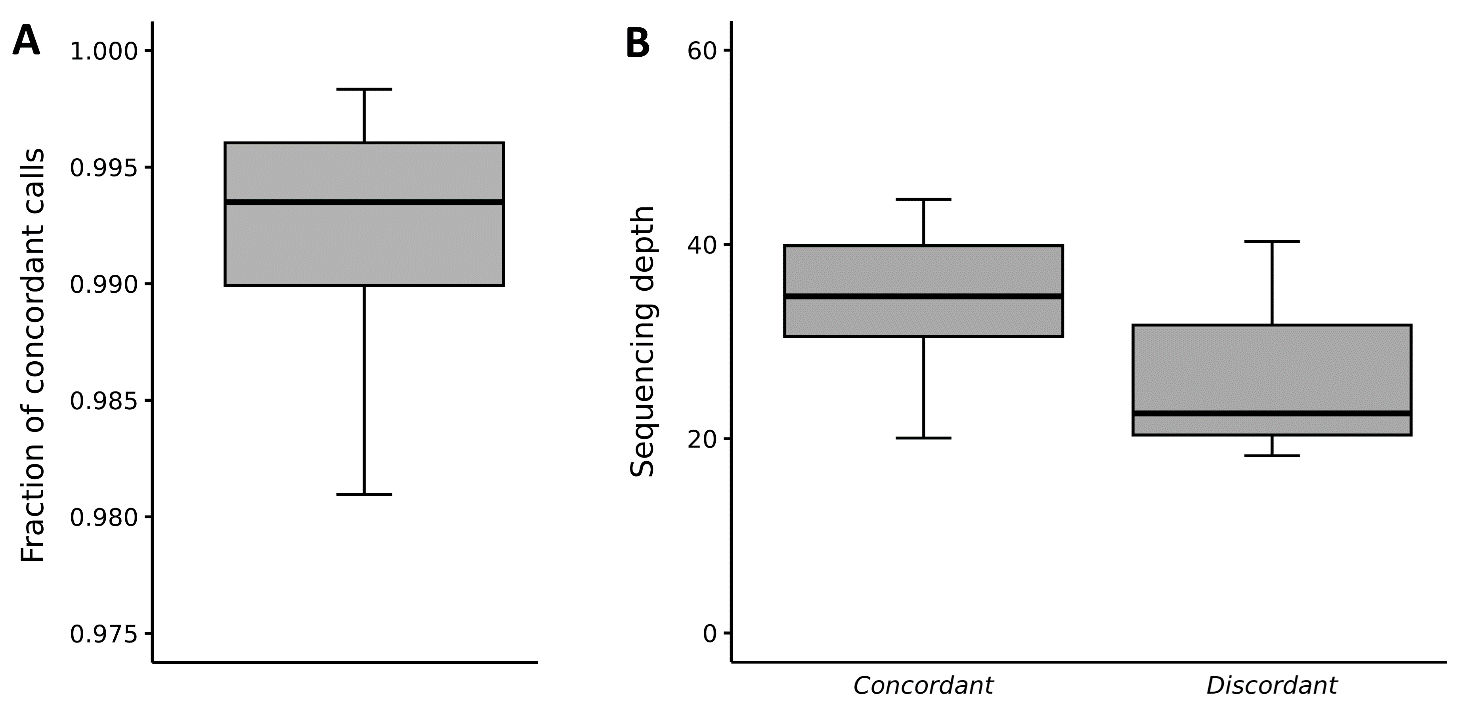


**Figure S10**. **A)** Fraction of concordant genotype calls between replicates. **B)** Average sequencing depth at concordant (left) and discordant (right) genotype calls.


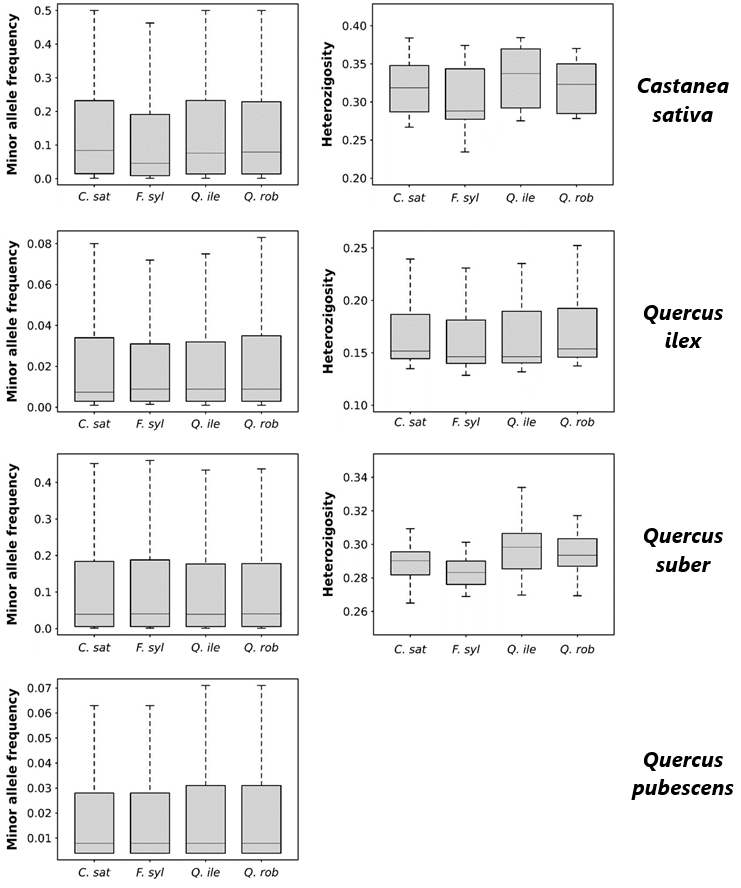


**Figure S11**. Comparison of genetic variability estimates obtained by stratifying the data based on the species for which the probes were designed.
